# A Relevant Screening of Organic Contaminants Present on Freshwater and Pre-Production Microplastics

**DOI:** 10.3390/toxics8040100

**Published:** 2020-11-09

**Authors:** Claudia Campanale, Georg Dierkes, Carmine Massarelli, Giuseppe Bagnuolo, Vito Felice Uricchio

**Affiliations:** 1 National Research Council, Water Research Institute (CNR-IRSA), 70125 Bari, Italy; carmine.massarelli@ba.irsa.cnr.it (C.M.); giuseppe.bagnuolo@ba.irsa.cnr.it (G.B.); vito.uricchio@ba.irsa.cnr.it (V.F.U.); 2 German Federal Institute of Hydrology (BfG), 56068 Koblenz, Germany; Dierkes@bafg.de

**Keywords:** PCB, PAH, DDT, non-target screening, Ofanto River

## Abstract

Microplastics (MPs) have recently been discovered as considerable pollutants of all environmental matrices. They can contain a blend of chemicals, some of them added during the manufacture of plastic to improve their quality (additives) and others adsorbed from the surrounding environment. In light of this, a detailed study about the identification and quantification of target organic pollutants and qualitative screening of non-target compounds present on MPs was carried out in different types of samples: environmental MPs, collected from an Italian river, and pre-production MPs, taken from the plastic industry. Polychlorobiphenyls (PCBs), organochlorine pesticides (OCPs), and polycyclic aromatic hydrocarbons (PAHs) were chosen as target compounds to be quantified by Gas Chromatography-Mass Spectrometry (GC–MS), while the non-target screening was carried out by High Resolution Gas Chromatography-Mass Spectrometry (HRGC–MS). The target analysis revealed concentrations of 16 priority Polycyclic Aromatic Hydrocarbons by Environmental Protection Agency (EPA-PAHs) in the range of 29.9–269.1 ng/g; the quantification of 31 PCBs showed values from 0.54 to 15.3 ng/g, identifying CB-138, 153, 180, 52, and 101 primarily; and the detected OCPs (p,p’-DDT and its metabolites) ranged between 14.5 and 63.7 ng/g. The non-target screening tentatively identified 246 compounds (e.g., phthalates, antioxidants, UV-stabilizers), including endocrine disruptors, toxic and reprotoxic substances, as well as chemicals subjected to risk assessment and authorisation. The large assortment of plastic chemicals associated with MPs showed their role as a presumable source of pollutants, some of which might have high bioaccumulation potential, persistence, and toxicity.

## 1. Introduction

Among the critical issues related to the high presence of microplastics in the environment, their potential role as an extra substrate for the partitioning and the diffusion of hazardous compounds is of great concern.

Microplastics (MPs) can sorb and concentrate persistent organic pollutants (POPs) six times higher than that found in the marine water due to their non-polar nature and elevate surface-area-to-volume ratio [1].

POPs such as organochlorine pesticides (OCPs), polychlorobiphenyls (PCBs), and polycyclic aromatic hydrocarbons (PAHs), which have been frequently reported [2,3,4,5,6,7,8,9,10,11,12], were found sorbed on plastic and MPs of marine environments. Moreover, inorganic pollutants have also been found [13,14,15]. As it is known, if organisms ingest MPs, these contaminants can transfer to food webs and cause consequences to human health [16,17] and ecosystems.

The sorption of hydrophobic pollutants to MPs through physical and chemical interactions is considered an essential environmental process because this will influence the mobility and bioavailability of these pollutants.

Changes such as pH, temperature, and ionic strength of the surrounding media can influence these processes [10,18,19]. Hydrophobic pollutants can pass through the polymer frame, depending on the dimension of pores composing the polymeric matrix and the molecular size of the chemical compound. Consequently, small molecules with lower weights will move more quickly through a polymeric structure with larger pores.

Depending on polymer type, its density and crystallinity, the surrounding media, and the kind of pollutants present, adsorption kinetics will change [8].

Plastic products comprising microplastics generally contain a potpourri of different chemicals introduced intentionally during manufacture to improve quality (additives) that can leach into the surrounding media during their use or their spread [20,21,22]. Moreover, many different types of plastic additives exist, including fillers, plasticisers, stabilisers, UV-antioxidants, flame retardants, biocides, pigments, and antistatic and conductive additives, among others [17,23]. Progressively more types of environmental contaminants have been discovered on plastic debris [16,24,25], but limited information about plastic additives as well as the nature and sources of chemicals included in MPs has been reported.

The poorness of information is partly because the elemental composition of plastic is very variable, and analytical methodologies to extract and analyse chemicals are still under development. A systematic overview of compounds and a screening of chemicals related to plastic manufacture is still missing [26].

For many years, most studies were conducted primarily on marine ecosystems. However, more recently researchers have started to expand their focus to include freshwater and terrestrial environments [27], demonstrating that rivers represent one of the principal routes for MPs to reach the oceans [28]. However, to date, few studies have considered the associations of hazardous compounds with MPs in freshwater ecosystems [29].

In light of this, our research was intended to determine chemical contaminants present in MPs collected in an Italian river in Southeast Italy (Ofanto River) in order to focus the attention on MPs as an important but often overlooked and underestimated vehicle of pollutants into the environment that can easily be ingested by organisms and be transferred in food webs. Some of these compounds originated from MPs themselves because of additives used during plastics production, while others are adsorbed from the surrounding environment. PCBs, OCPs, and PAHs have been chosen as target compounds to be quantified as environmental organic pollutants. At the same time, a qualitative non-target screening was carried out by High Resolution Gas Chromatography-Mass Spectrometry to identify a profile of emerging micropollutants carefully.

We were also interested in studying pollutants separately in a different pool of MPs. Some of the pollutants were collected from the river (environmental origin), and the others were obtained from a local plastic industry that uses virgin pre-production materials that should have fewer contaminants compared to environmental samples.

## 2. Materials and Methods

### 2.1. Samples Collection

#### 2.1.1. Environmental Microplastics

Microplastics were sampled from Ofanto, the most significant river in the Apulia region (South Italy), which is 165 km long, with a water flow of 15 m^3^/s and a catchment area of 2790 km^2^. Five seasonal sampling campaigns were planned, aiming to monitor the variation of MP concentrations over a year. River surface samples were collected during February, April, October, and December 2017 and May 2018, being taken 6 km from the Ofanto river mouth (41°17′20.22′’N; 16°06′92′’E).

Aiming to decrease the spatial and temporal variability [30], we sampled microplastics by 3 plankton nets (2.5 × 0.55 m) of 333 μm mesh size fixed in the middle of the river at the same time for 2 different time bands (11:00–13:00 and 13:00–15:00), collecting 6 replicates for each campaign for a total of 20 samples. The net was placed with a portion of it kept out of the water, filtering the first 45 cm of river surface. The portion of the net underwater was registered, ensuring a constant submersion for all the sampling. At the end of sampling time, we washed each net from the outside to the inside with deionised water, conveying all the sampled material (plastics and microplastics, natural debris, sediment, and other materials) into the collector tube attached to the tail of the net.

Material captured by trawls was differentiated through 5000 µm and 300 µm stainless steel sieves. Material retained on the 5.0 mm sieve was disposed of while that of the 300 µm sieve was placed in a 35 °C drying oven for 24 h until to sample dryness. The biggest plastic particles (5000–2000 µm), visible to the naked eye, were randomly selected and isolated from each sample and the particles picked manually using forceps were grouped into five mean seasonal samples. The extracted microplastics were then placed in sterile plates and stored until chemical investigations.

Further details inherent sampling strategy and methodologies are described in [29].

#### 2.1.2. Pre-Production Microplastics

Three main groups of pristine pre-production microplastics were also provided and analysed together to environmental samples to compare the amount of adsorbed environmental contaminants to the concentration of contaminants originating from plastics:

- virgin colourless polyethene (PE) pre-production pellets (size 5000 µm);
- virgin green polyethene (PE) pre-production microparticles (size <500 µm);
- virgin colorless polypropylene (PP) pre-production pellets (size 5000 µm).

These virgin pre-production materials were purchased from a local plastic industry.

#### 2.1.3. Water Samples

In order to compare pollutants concentration associated with MPs to contaminant concentration found in the river, we collected three replicates of water samples during the May 2018 campaign. Samples were collected in dark glass bottles and stored at 4 °C until extraction. 

### 2.2. Chemical Identification

The chemical identification of the different polymers was performed by Pyrolysis–GC–MS, as reported in [29]. About 3% of MPs (chosen on the basis of the overall morphology of particles found in samples) were hand-picked from each sample and flash-pyrolysed at 600 °C, cutting ≈10–30 μg of plastic material from each particle. The separation of pyrolysis products was performed by gas chromatography using a 7890B gas chromatograph (Agilent Technologies, Santa Clara, CA, USA) equipped with a 30 m Ultra ALLOY-5 capillary column (Frontier Lab, Koriyama, Japan). The temperature of the GC oven was initially set at 40 °C and held for 2 min, secondly enhanced with 20 °C min^−1^ until 320 °C, and finally held constant at 320 degrees for 13 min. Identification of products was detected by high-resolution mass spectrometry using a 7200 Q-ToF (Agilent Technologies, Santa Clara, USA) operating in electron ionisation (70 eV) and full scan (*m*/*z* 50–500) mode. Specific products of pyrolyzation were selected as indicators for qualitative polymer analysis to characterise polymers unequivocally [29]. 

#### 2.2.1. Pollutants Extraction from Microplastics Samples

The pollutant extraction procedure was selected on the basis of the physicochemical features of the target analytes to be analysed, an analysis of the extraction procedures already tested in previous studies on MPs [5,16], the costs, and the available resources.

About 250 mg of MPs for each mean seasonal sample (environmental sample) and the same amount for the virgin samples were homogenised with diatomaceous earth forming a free-flowing powder. Then, samples were spiked with known concentrations of an internal standard solution of the labelled compound [13C12]PCB 104. 

The material was extracted using 5 mL of n-hexane until complete submersion of the sample; this was followed by sonication (Branson 5210R-MT Ultrasonic Cleaner) for 30 min at 40 °C. The complete extraction process was repeated 2 more times with 2 additional 5-mL portions of clean solvent to guarantee full extraction of analytes. The extracts were then concentrated until incipient dryness under gentle nitrogen stream (using a Caliper Life Sciences TurboVap II Concentration Workstation) and re-solubilised into 0.5 mL nonane. The validation of extraction procedure was checked, before determination of real samples, spiking a known amount of natives’ analytes on the three main groups of virgin pre-production microplastics (previously described in Section 2.2) in order to simulate environmental samples. The recovery of the internal standard consisted of a mean of 75% (Table S4). The recoveries of the target analytes in the matrix-spiked blanks (pre-production microplastics) were acceptable, consisting of a mean of 70% (Table S5). Further details regarding QA/QC measures are provided in Supplementary Materials.

#### 2.2.2. Pollutant Extraction from River Water Samples

Every sample was spiked with noted concentrations of internal standard and extracted with a liquid–liquid method; 100 mL of n-hexane was added to 600 mL of samples and agitated for 3 h.

For sample cleanup, 50 mL of extract was loaded into cartridges with 15 g of anhydrous sodium sulfate (BondElut Agilent) and eluted with 10 mL of hexane.

The extracts were then concentrated using a gentle nitrogen stream (using a Caliper Life Sciences TurboVap II Concentration Workstation) and re-solubilised into 0.5 mL nonane.

### 2.3. Target Compound Analysis by GC–MS/MS

All microplastic samples (Table 1) were analyzed by GC–MS/MS in order to find the following anthropogenic contaminants: 31 PCBs (CB#18, 28, 52, 44, 95, 101, 99, 81, 77, 110, 151, 123, 149, 118, 114, 146, 153, 105, 138, 126, 187, 183, 128, 167, 177, 156, 157, 180, 169, 170, 189), 8 OCPs (alfa-Hexa-Chloro-Cyclohexane, beta-Hexa-Chloro-Cyclohexane, gamma-Hexa-Chloro-Cyclohexane, delta-Hexa-Chloro-Cyclohexane, aldrin, p,p’-Dichloro-Diphenyl-Trichloroethane, p,p’-Dichloro-Diphenyl-Dichloroethane, p,p’-Dichloro-Diphenyl-Dichloroethylene), and 16 EPA-PAHs (acenaphthylene, acenaphthene, fluorene, phenanthrene, anthracene, fluoranthene, pyrene, benz(a)anthracene, chrysene, benzo(b)fluoranthene, benzo(k)fluoranthene, benzo(a)pyrene, indeno(123-cd)pyrene, dibenzo(ah)anthracene, and benzo-(ghi)perylene). The analyses were performed using a ThermoElectron TRACE GC Ultra coupled with a PolarisQ Ion Trap (Thermo Electron, Austin, TX, USA) mass spectrometer equipped with a Programmable Temperature Vaporizing (PTV) injector and a TriPLUS^TM^ RSH^TM^ autosampler. The system was managed by Thermo Electron Xcalibur software version 1.4.1. The compound separation was achieved using an Agilent CP8944 VF- 5ms U (length 30 m, i.d. 0.25 mm, film thickness 0.25 µm) column. More details regarding chemical standards and solvent materials, sample preparation, chemical analyses, instrumental analyses, precursor and product ions (Tables S1–S3) are described in the Supplementary Information. Total Ion Current Chromatograms for each group of pollutants are shown in Figures S1–S4. 

### 2.4. Non-Target Compounds Screening by GC–HRMS

A non-target qualitative screening of contaminants present on environmental MPs was carried out by gas GC–HRMS analyses using a 7890B gas chromatograph (Agilent Technologies, Santa Clara, CA, USA) equipped with a 30 m J&W HP-5MS (5% phenyl methyl siloxane) capillary column with an inner diameter of 250 µm and a thickness of 0.25 µm coupled to a 7200 Q-ToF (Agilent Technologies, Santa Clara, CA, USA) working in electron ionisation (70 eV) and full scan (*m/z* 50–500) mode. The carrier gas used was helium (flow of 1 mL/min). The GC oven temperature program was 80 °C (held 2 min)-170 °C-30 °C/min-320 °C-12 °C/min (held 10 min). Two microliters were injected in the splitless mode, and EI mass spectra were analysed in the high-resolution mode (>25,000 resolution).

Compound identification was performed with Agilent MassHunter Unknowns Analysis B.08.01 using the SureMass algorithm for data processing [31].

High-resolution MS profile data were extracted and converted in SureMass Chromatograms. Only peaks with a relative area greater than 1% of the largest peak were selected, and relative compounds were identified for comparison of the mass spectra with the NIST 17 library, adopting a minimum similarity criterion of 70%. We refer to the latter compounds as tentatively identified as we did not use authentic standards to confirm their identity. For each chromatogram, we performed a blank subtraction.

## 3. Results and Discussion

This research allowed us to identify an extensive profile of plastic-related chemicals such as different environmental pollutants (PCBs, PAHs, OCPs) next to a variety of additives, antioxidants, degradation products, and biofilm compounds in environmental MPs collected from a river and in a different typology of virgin pre-production MPs. 

The chemical characterization of environmental MPs revealed a prevalence of polyethylene (PE) (76%) particles collected, followed by polystyrene (PS) (12%), polypropylene (PP) (10%), polyvinylchloride (PVC) (0.7%), and polyurethane (TDI-PUR) (0.35%) items. This polymer distribution was probably related to the main source of MPs in the Ofanto River linked to agricultural practices [29] that mainly use PE film for mulching [32].

### 3.1. PCBs

All environmental MP samples contained PCBs (expressed as the sum of 31 congeners) in the range from 0.54 to 15.3 ng/g with a mean value (calculated as the average of four sampling campaigns) of 12.89 ± 3.4 ng/g.

The most significant concentrations were observed in environmental MPs with values measured ranging from a maximum of 15.3 ng/g, found in samples of May 2018, and a minimum of 7.87 ng/g observed in December 2017.

**Figure 1:**
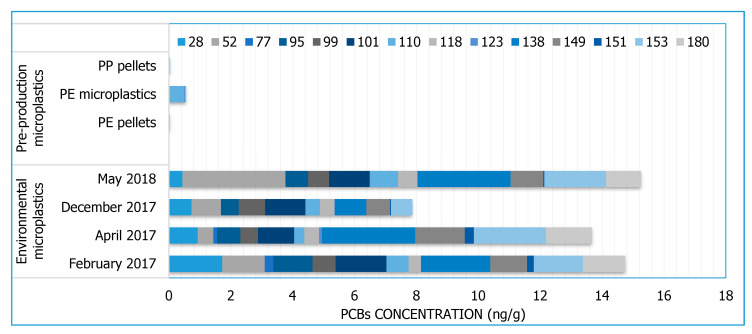
Concentrations of 14 polychlorobiphenyl (PCB) congeners, expressed as ng/g, registered on environmental MPs (April 2017, February 2017, December 2017, May 2018) and on virgin microplastics (MPs) (virgin polyethene (PE), virgin coloured PE, virgin polypropylene (PP)). Virgin PE and PP were colourless pellets while virgin coloured PE was green microparticles (<500 µm).

A total of 14 PCB congeners (CB-28, 52, 77, 95, 99, 101, 110, 118, 123, 138, 149, 151, 153, 180) out of 31 investigated were measured in a concentration above the detection limit (0.2 ng/g), and 3 of these (52, 138, 153) reached the highest concentrations (Figure 1). These congeners were identified by the Stockholm Convention on Persistent Organic Pollutants (POPS) as three of the six indicator PCBs (CB-28, CB-52, CB-101, CB-138, CB-153, and CB-180) to characterise contamination by PCBs. 

The amount of total PCBs found on pre-production MPs was reasonably lower than the concentration observed on microparticles sampled from the river, and only two individual PCBs (PCB-110 and 153) were revealed as sorbed on virgin particle debris. This can be explained because PCBs are generally associated with environmental contamination and are not intentionally added during the production of plastic products (as occurs in the case of some hydrocarbons), which is why they were not detected in virgin MPs.

The concentrations of each PCB congener detected in all samples are summarised in Tables S7 and S8.

Although the ban on the use of PCBs in Italy since 1983 [33] (Directive 76/769/EEC) and their total absence revealed in water samples analysed probably due to their lipophilicity, we still found countable amounts of PCBs registered on environmental MP samples, confirming that they can concentrate, transport, and spread these pollutants from their sources to the marine environment throughout rivers.

Plastic waste and microplastics were found to be suitable substrates for concentrating POPs from areas polluted by these contaminants [2,3,34,35,36,37]. PCBs, are the most persistent among POPs. When they are in sediment or surface water, they tend to be adsorbed by hydrophobic interactions between non-polar (or slightly polar) molecules and the non-polar surface of particulate materials or sediment particles [38], with microplastic not representing an exception. 

Our data are comparable to those reported by other authors on PCBs sorbed on MP debris from seawaters all over the world [39,40,41]. However, other authors have reported higher concentrations of PCBs than ours, up to 223, 307, 980, 2285.8, and 7554 ng/g^−1^ detected by [2,39,42,43,44], respectively. This variability can be explained by a possible shorter dwelling time of our MPs into the environment, with rivers being one of the starting forms of transport for MPs to reach the oceans. Studies estimated that 80% of MP pollution in the seawater comes from the land [45]. 

Moreover, some authors related the time of permanence of microplastics into the environment to a more significant amount of organic pollutants detected on particles. This is explained by the higher level of ageing of particles that increase the sorption of chemicals [36,39].

### 3.2. PAHs

**Figure 2:**
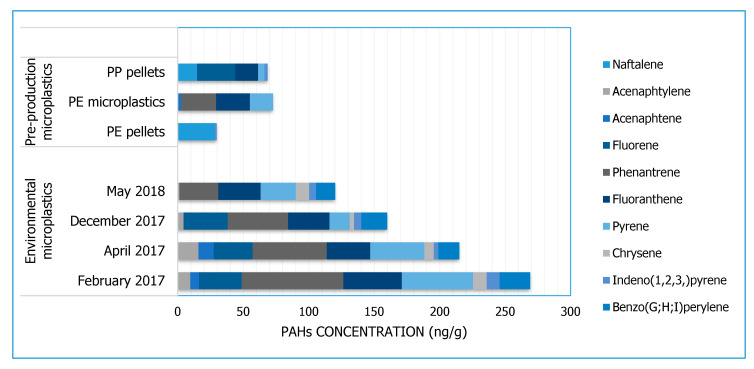
Concentrations of 16 EPA-PAHs, expressed as ng/g, registered on environmental MPs (April 2017, February 2017, December 2017, May 2018) and on virgin MPs (virgin PE, virgin coloured PE, virgin PP). Virgin PE and PP were colourless pellets while Virgin coloured PE was green microparticles (<500 µm).

All samples, except river water samples, were found to contain PAHs. Their quantities are reported in Figure 2. The sums of all observed PAHs (classified as priority pollutants by the United States Environmental Protection Agency, USEPA) were in the range of 29.9–269.1 ng/g, with a mean value of 191.1 ± 64.9 for environmental samples and 57.1 ± 23.7 ng/g for virgin MPs. The largest concentration of hydrocarbons was found in environmental MPs collected from the river in February and April 2017 (269.1 ng/g and 215.1 ng/g, respectively) followed by December 2017 (160.1 ng/g) and May 2018 (120.3 ng/g). Conversely, transparent pre-production PE pellets revealed the lowest contents of PAHs for a total concentration equal to 29.9 ng/g, while in coloured pre-production PE MPs (72.8 ng/g) and pre-production PP pellets (68.7 ng/g), PAH values were twice as high. The PAHs acenaphtylene, phenanthrene, fluoranthene, pyrene, chrysene, indeno(1,2,3,)pyrene, and benzo(G;H;I)perylene were detected in all samples (April, February, December, and May). Acenaphtene was only identified in April and February 2017, while fluorene was found in all campaigns except for the month of May. Naphthalene was present only in pre-production PE and PP pellets. The remaining PAHs were found in concentrations below the detection limits (Table S6). River water samples showed values below the detection limit for all PAHs investigated.

The International Pellet Watch program suggests the use of aged PE pellets as a monitoring tool for investigating the presence of pollutants adsorbed on pellets released in the marine environment [46]; the monitoring of PAHs detected the amount of hydrocarbons ranging from concentrations lower than the limit of quantification (Table S9) to values greater than 24,000 ng/g-pellets, with an average of about 3000 ng/g-pellets. 

Similarly, a study conducted by [47] revealed the presence of PAHs adsorbed on MPs sampled in the Feilaixia Reservoir (Guangdong Province, China), with a total concentration of 16 PAHs ranging from 282.4 ng/g to 427.3 ng/g.

These results are greater than those detected in the present study (30–270 ng/g), even if there are no Italian studies on marine pellets and limited studies on MPs of freshwater environments to be compared to.

The PAH composition found on samples analysed in the present work revealed a similar distribution of hydrocarbons in all MP environmental samples, showing a prevalence of pheanantrene followed by fluoranthene and pyrene. Although our analyses on virgin pre-production MPs were limited to only two polymers (colored/colorless PE, and PP), the results revealed the presence of different PAHs, such as naphthalene, fluoranthene, acenaphthene, pyrene, and phenanthrene, as already reported by [27].

**Figure 3:**
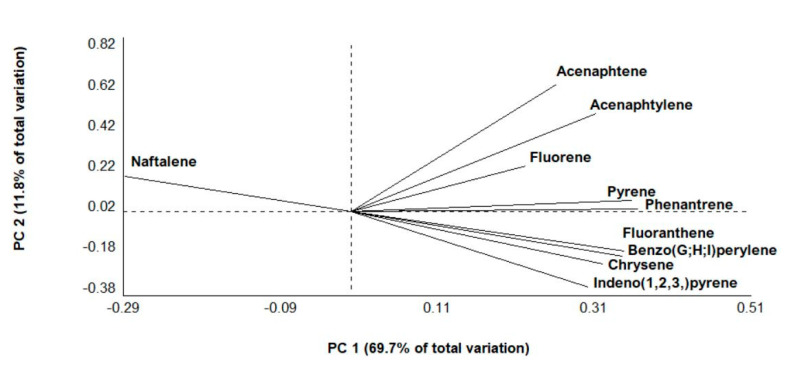
Principal component analysis (PCA) considering the relative higher abundance of the PAHs, grouped according to the similarity.

The differences in the distribution of PAHs between environmental and virgin MPs were analysed by principal component analysis (PCA), considering eigenvectors >0.5 as the most significant contribution. The analysis showed that the first principal component (PC1) explained 69.7% of the data variability, while the second component (PC2) accounted for 11.8% (Figure 3). The differences in the distribution of PAHs created two significant groups and one smaller group. Group I was due to the presence of naftalene found only in virgin transparent PE and PP pellets, suggesting that low molecular weight hydrocarbons are less sorbed on MPs than high molecular weight compounds, probably because they are more volatile and quickly degraded [48]. Acenaphtene, Acenaphtylene and Fluorene compose Group II because found predominantly in environmental MPs than in virgin ones and Pyrene and Phenanthrene Group III due to their presence in environmental MPs and on virgin coloured PE. It is no coincidence that the physical properties of microplastics (such as colour, shape, and size) not only influence hydrocarbons concentrations but can also alter their composition on plastic [48]. Indeed, the authors of [48] demonstrated that darker pellets tend to contain higher weight hydrocarbons than lighter coloured pellets.

In previous studies, PAHs found sorbed on macro- and microplastics have been associated with petrogenic or pyrogenic sources.

Depending on specific ratios of individual or methylated PAHs, it is possible to go back to their sources such as oil seeps or combusted fuels [2,42,44,45,49].

However, attention should be paid when calculating PAH ratios for plastic, as these indices are usually calculated for source determination of PAHs in sediment [49]. Additionally to the environmental contamination (e.g., given by oil seeps or combusted fuels), an indefinite amount of PAHs found in the current study could be related to the plastic itself, as we found them both on environmental and virgin MP samples. Indeed, these pollutants have also been found previously by other authors on unpolluted plastic such as baby feeding bottles [50] and on virgin pellets [27].

### 3.3. OCPs 

Regarding the group of organochlorine pesticides, we found that just four of them were present in environmental MPs samples (p,p-DDT, p,p-DDD, p,p-DDE, gamma-HCH).

**Figure 4:**
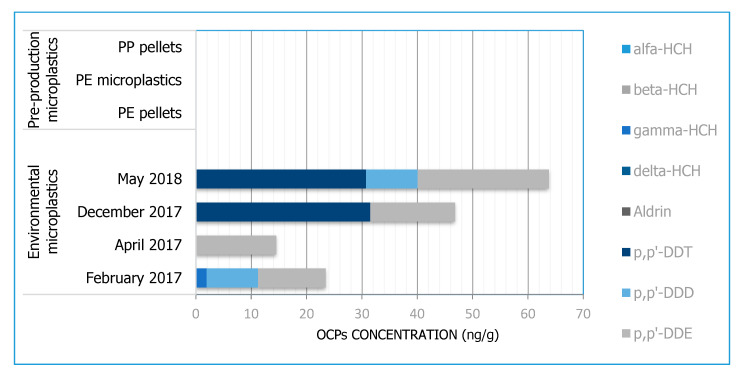
Concentrations of eight organochlorine pesticides (OCPs), expressed as ng/g, registered on environmental MPs (April 2017, February 2017, December 2017, May 2018), and on virgin MPs (virgin PE, virgin coloured PE, virgin PP). Virgin PE and PP were colourless pellets while virgin coloured PE was green microparticles (<500 µm).

The pollutants belonging to the DDT group (DDT, DDD, DDE) reached the most significant amount in environmental MPs, with a mean value of 37.1 ± 22.4 ng/g. The p,p’-DDT resulted in the compound with the highest concentrations, although it reached measurable levels of 31.53 and 30.75 ng/g only in the samples collected in Ofanto River during December 2017 and May 2018 campaigns, respectively. DDTs transformation products p, p’-DDD, and p,p’-DDE were also detected—the former two in February 2017 (9.29 ng/g) and May 2018 (9.33 ng/g) campaigns, respectively, and the latter in all sample campaigns exceeding the Italian limit of 10ng/g fixed for soils (D.Lgs 152/2006), with values ranging from 12.2 ng/g (February 2017) to 23.7 ng/g (May 2018) (Figure 4). DDT represents the original compound released to the environment, but it often decomposes to DDD and DDE, the two main products of dechlorination of DDT by microorganisms or abiotic degradation [51,52].

DDE concentrations on PP MPs collected from four coastal areas in Japan revealed values ranging from 0.16 ng/g to 3.1 ng/g [1], and the International Pellets Watch reported the highest concentrations of DDTs (DDT, DDD, and DDE) in Hermosa Beach, CA, USA (267 ng/g), as well as concentrations of 4.49 ng/g and 2.43 ng/g in the Bay of Maputo, Mozambique, and South Durban, South Africa, respectively [53]. 

River water samples were also collected and analysed simultaneously with MP debris in order to compare the concentration of contaminants adsorbed to the concentration of pollutants found in the surrounding environment; values above the detection limit were not found in any samples. 

The levels of DDT and its degradation products (DDD and DDE) detected on environmental MPs are much higher than previous values (2016–2017) recorded in surface waters and sediment during the monitoring campaigns in Ofanto River by Regional Agency for Environmental Prevention and Protection (ARPA Puglia) [54]. 

These values suggest that DDT could have been adopted as a pesticide in that area, probably in previous years when it was legal. Indeed, Apulia is well-known for its vast vineyards and olive plantations throughout the whole territory [55,56]; high DDT residues may originate from agricultural activities in these areas [57].

The total production of DDTs reached 3 million metric tons in the previous century, and its consumption rate in Mediterranean area achieved approximately 2000 t/year [58].

OCPs are persistent compounds, and they have a high affinity for organic matter due to their non-polar nature; therefore, a remarkable quantity of these pesticides may remain stored in soils [59]. 

Increasing the input of MPs in soils due to their common terrestrial origin is of crucial importance to consider their ability to bind chemicals compared to other compartments of the soil. 

The soil, then, could become a remission source because MPs can be transported by washout and surface runoff into surface waters, as well as via leaching into groundwater through irrigation [60,61].

Our results show that MPs play a role in aquatic ecotoxicology as vector and concentrator of these toxic substances, becoming part of the aquatic ecosystem. Although the bioavailability of these pollutants carried by MPs has not been studied in detail, MPs considerably increase the complexity of monitoring water pollution’s interaction with the entire ecosystem in different ways.

Nowadays, regulations about surface water monitoring do not consider MPs and their interactions with pollutants, which results in an emerging and complex issue that underlines the need for a deepening and revision of current legacy.

Except for gamma-HCH, which showed measurable levels only in MPs in the February 2017 campaign (Table S10), all the others pesticides investigated revealed concentrations below the detection limit in environmental MPs, such as virgin pre-production MPs and river water samples.

### 3.4. Non-Target Screening

General screening of organic compounds detected on environmental MPs (collected in April, February, and December 2017 and May 2018 campaigns) revealed the presence of a broad range of compounds (Figures S5–S8), 248 of which were identified by comparison with the spectra present in the NIST 17 library (Table S13), with a match factor ≥70% (Table S11). Of these, 37 were identified with a ≥ 80% confidence match factor and are summarised in Table 2.

Fifteen unknown plastic related compounds, identified for comparison by spectra of NIST 17 library with a match factor ≥85%, were further confirmed on the basis of accurate mass measurements calculating mass errors related to the major ions observed for each compound (Table S12).

In Table 2, organics tentatively identified are sorted on the basis of the primary source of origin: plastic additives (UV stabilisers, phthalathes, antioxidants); hydrocarbons (alkanes, alkenes, substituted and cyclic hydrocarbons); intermediate; alcohol; biofilm and algae compounds. 

The HRGC–MS screening identified a broad range of plastic additives. These compounds are linked to plastic production processes and are known to be added intentionally to plastic to make it soft and flexible, or they are degradation products.

Several different types of additives are used in plastic manufacture, including fillers; pigments; plasticizers; UV stabilizers; antioxidants; antiozonants; flame retardants; anti-degradants; slip stabilizers; lubricants; optical brighteners; antifog, antistatic, and conductive additives; food contact; and medical additives [17,23,79].

Most of these are not chemically bound in plastics but can migrate, i.e., when chemical compounds present in the plastic structure move to its surface or to the surroundings in contact with the item. 

Superficially, the compound may evaporate or be removed, e.g., washing or contact with human skin. The capability to migrate significantly influences the potential for release of compounds from plastics, and thereby the potential for exposure of consumers [79]. Both phthalates and flame retardants are substances that are well known to migrate. 

The risks for human health of other plasticisers (often used as substitutes for phthalates) are not always evident, due to limited toxicological data. The level of plasticisers in plastic can reach up to 50% by weight. Thus, it makes it possible that a significant quantity can leach when plastic comes in contact with moisture. For these reasons, the entire group of plasticisers can be considered as a hazard category [63].

Plasticisers are often connected to polyvinyl chloride, although they have also been identified in printing inks and lacquers employed in the manufacturing of such other polymers [80].

Phthalates, UV-stabilizers, and antioxidants were the dominant classes of additives identified in this screening.

Among additives, phthalates, represent a category strongly related to plastic and MP debris. Indeed, they have been used in previous studies as an indicator of the presence of plastic waste in skin biopsies of fin whales of the Mediterranean region that were chronically exposed to these contaminants due to plastic and microplastic ingestion [81,82].

Different phthalates were found in this screening including two (dibutyl phthalate and bis(2-ethylhexyl) phthalates of the five phthalates (bis(2-ethylhexyl) phthalate (DEHP), diisononyl phthalate (DINP), dibutyl phthalate (DBP), benzyl butyl phthalate (BBP), and di-n-octyl phthalate (DNOP)) mentioned in the Commission Decision 1999/815/EG. Phthalates have undergone risk assessment by the EU, and they have been relatively thoroughly investigated for their effects on the environment and human health as well as their use in different types of products.

Indeed, they can be endocrine disruptors, toxic to reproduction, or even suspected carcinogens (Table 2) [17,72,83,84,85,86]. Compared to other endocrine disruptors (EDCs), phthalates are particularly concerning because of their extensive presence, including their high levels in the environment [87,88,89,90,91,92]. Phthalates were also identified in domestic wastewater, evidencing their leeching from plastic products into the surrounding ambience [90,91,93].

Dibutyl phthalate, identified in this screening, is mainly related to PP and PE products and has also been previously identified in marine debris [63,94,95].

Significant quantities of bis(2 ethylhexyl) phthalate (DEHP) have also been found in artificially aged rubber toys in comparison to low concentrations in unaged toys [96], suggesting a probable release of these substances during the weathering process.

DBP and Diethyl phthalate (DEP) are phthalates most broadly used in medicines, even if toxicological effects have been observed in animals. These findings have clinical relevance and cannot be ruled out; thus, the European Medicines Agency (EMA) will probably establish limits for the use of DBP in medicines. Furthermore, the agency will probably also establish limits for the use of DEP and Polyvinyl acetate phthalate (PVAP) in medicines [72].

The sorption of DBP and DEP on microplastics was studied in a study conducted by [97], wherein the authors demonstrated that the hydrophobic interaction and ionic strength dominated the partition, with the sorption of DBP being almost 100 times higher than DEP. Moreover, solution pH and natural organic matter had small impacts on phthalate sorption by microplastics, indicating that microplastics could accumulate them in different aquatic environments. 

Because of the potential risk of DEHP and DBP [98,99,100,101] and the potential hazard of other phthalates, this group is considered a hazard category [63].

Together with phthalates, several types of antioxidants were also found in the environmental microplastic samples. They are compounds that work to slow down the oxidation cycle, usually by scavenging free radicals to inhibit the oxidative degradation of plastics. Oxidation during mixing or manufacture can cause problems such as loss of strength, breakdown, or discolouration; oxidation can also verify in the final product causing discoloration; scratching; and loss of strength, flexibility, stiffness, or gloss [63]. 

Many antioxidants can be used to prevent the ageing of plastic, such as phenolic antioxidants, organophosphorus compounds, and hindered amines and thioesters [102]. In this screening, bumetrizole, 1,4-benzenediamine, N-(1-methylethyl)-N’-phenyl- (commercially known as Santoflex IPPD) and Tris (2,4-di-tert-butylphenyl) phosphate (Irgafos 168) were found. Bumetrizole is generally used to slow the oxidation process of the polymer exposed to UV light. It preferentially degrades itself and helps in this way to stabilise the polymer [71,72]. It does not have high toxicity, but it does not allow for contact with fatty foods. 

Antioxidants are used in little quantities up to 2% *w/w* (20,000 mg/kg or ppm) in almost all commercial polymers.

Among additives, stabilisers were also detected in this screening. Generally, stabilisers are used to prevent plastic’s degradation (e.g., by temperature, light, UV light, oxygen, and other types of weathering), thus prolonging the lifetime of the products. 

Heat stabilisers have a role in protecting synthetic polymers during the thermal production process to avoid product decomposition and to protect against heat in long-term use at high temperatures. 

UV stabilisers are used to prevent or protect degradation of plastics from ultraviolet rays by absorbing the harmful UV light that changes the physical and optical properties of the polymer, protecting the plastic against discolouration, cracking, brittleness, or other loss of desirable physical properties. The stabilisers act in such a way that they will be decomposed instead of the plastic polymers [79,103]. This behaviour means that for mechanical recycling of plastics, it is necessary to add further stabilisers for protection against degradation in the newly recycled plastic products [79].

Typical UV stabilizers are benzophenones, hindered amines, and benzotriazole. Salicylate esters, cyanoacrylates, and benzylidene are also used, but are not as effective [104]. The following six UV stabilisers were identified in extracted samples: octabenzone (Uvinul 3008); 2-propenoic acid, 3-(4-methoxyphenyl)-, 2-ethylhexyl ester; benzophenone; phenol, 2-(5-chloro-2H-benzotriazol-2-yl)-4,6-bis(1,1-dimethylethyl)-; 2,4-di-tert-butylphenol; and benzenepropanoic acid, 3,5-bis(1,1-dimethylethyl)-4-hydroxy-octadecyl ester (Irganox 1076).

Many additives such as benzotriazole UV stabilisers and phenolic antioxidants are expected to have log Kow (log n-octanol/water partition coefficient) values between 5 and 8 and have the potential for bioaccumulation. Although field observations are lacking information related to environmental partitioning of these chemicals, their metabolic transformation, and biodegradability, on the basis of estimated high partition coefficients and low biodegradability, it is possible to hypothesise their concentration through the marine food chain.

Experimental studies of phenolic antioxidants and benzotriazole UV stabilisers in sediments and fish partly support this hypothesis [105,106,107], even if it is not ensured that plastic waste is a relevant source of these compounds in the environment [79,108]. Octabenzone is a UV absorber/screener used to protect polymers (e.g., polyethene, polypropylene, polyvinylchloride) against damage by UV light and is subjected to risk assessment and authorisation before use [69]. Octyl methoxycinnamate (trade names Eusolex 2292 and Uvinul MC80) is a UV absorber and also a Federal Food, Drug, and Cosmetic Act (FDA) category 1 sunscreen, approved worldwide at concentrations up to 10%, and is the most frequently used sun screening agent. 

## 4. Conclusions

The present data represent a detailed survey of pollutants investigated on different types of MP samples—some collected in a freshwater environment, others provided by a plastic industry as virgin materials. This research provides one of the few datasets of chemicals associated with MPs in a freshwater ecosystem. Almost always, environmental MPs showed greater values of pollutants than virgin ones. PAHs resulted in the category with higher concentrations, with a mean value of 191.1 ± 64.9 for environmental samples and 57.1 ± 23.7 ng/g for virgin MPs. The DDT group was detected only on environmental MPs, showing concentrations higher than the Italian regulatory limit set for soils. Similarly, PCBs were detected almost exclusively in environmental samples, with concentrations ranging from 7.9 to 15.3 ng/g.

In general, lower concentrations of PCBs, PAHs, and OCPs were detected in our MPs collected in a freshwater ecosystem in comparison with contaminants found sorbed on samples collected in marine environments in previous studies. This is probably explained because rivers are one of the starter pathways of MPs, implying a briefer time of permanence in the freshwater environment with respect to the oceanic zones that are, on the contrary, the final receptors. The consequence is a different level of pollution between the two environments given by the lower level of ageing of MPs collected in rivers that is known to be a factor responsible for the sorption of pollutants. 

The non-target screening of freshwater MPs provided evidence of a pool of chemicals associated with MPs, and more than 200 organic substances have been identified, (UV stabilisers, phthalates, antioxidants, hydrocarbons, lubricants, intermediates, biocides, biofilm compounds, and degradation products), pointing out that MPs represent a multifaceted stressor for ecosystems. These results underscore the role of MPs as a multifaceted stressor for ecosystems because on the one hand they are themselves a source of a broad range of chemicals used as additives during plastic production, and on the other hand pollutants belonging from other sources and spread in the environment are sorbed on microplastics.

## Figures and Tables

**Table 1:** Details of microplastic samples and pollutants detected for target compounds analysis by Gas Chromatography–Tandem Mass Spectrometry (GC–MS/MS).

**Pollutants Analysed**	PAHs	Naphthalene, acenaphtylene, acenaphtene, fluorene, phenanthrene, anthracene, fluoranthene, pyrene, benzo(a)anthracene, chrysene, benzo(b)fluoranthene, benzo(k)fluoranthene, benzo(a)pyrene, indeno(123)pyrene, dibenzo(ah)anthracene, benzo(ghi)perylene	
	OCPs	Alfa-HCH, beta-HCH, gamma-HCH, delta-HCH, aldrin, p,p’-DDE, p,p’-DDD, p,p’-DDT	
	PCBs	18, 28, 52, 44, 95, 101, 99, 81, 77, 110, 151, 123, 149, 118, 114, 146, 153, 105, 138, 126, 187, 183, 128,167, 177, 156, 157, 180, 169, 170, 189	
**Microplastics Analysed**	Pre-production microplastics	PE transparent pellets (size 5000 µm) PP transparent pellets (size 5000 µm) PE green microplastics (size 300 µm)	
	Environmental microplastics	February 2017	Polymer: PE 77%, PS 13%, PP 10% Morphology: fragments 50%, pellets 30%, lines 10% Color: transparent 50%, black 40%, colored 10% Size: 5000–1000 µm
		April 2017	
		December 2017	
		May 2018	

**Table 2:** Overview of 37 different types of compounds tentatively identified on environmental microplastic samples (April 2017, February 2017, December 2017, May 2018) by general screening. The chemicals are grouped according to their presumed source. CAS no. and NIST probability match factor % for each compound is shown, along with molecular weight and chemical formula. Where available, common name and comments also are given; otherwise n/a is stated.

Environmental Microplastics						
Category	CAS#	Compound Name	Match Factor	Formula	Library Molecular Weight	Common Name and Comments
Hydrocarbons/ alkanes and substituted hydrocarbons	544-76-3	Hexadecane	96.33	C_16_H_34_	226.266	n/a Originate from the paraffin wax used as an external lubricant in PVC and other polymers. Alkanes are also used as a solvent and are oligomers originating from poly olefines (PP, PE, and PS) during recycling [62,63].
	593-49-7	Heptacosane	94.68	C_27_H_56_	380.438	
	630-04-6	Hentriacontane	93.29	C_31_H_64_	436.501	
	630-02-4	Octacosane	91.34	C_28_H_58_	394.454	
	1186-53-4	Pentane, 2,2,3,4-tetramethyl-	92.25	C_9_H_20_	128.157	
	62108-23-0	Decane, 2,5,6-trimethyl-	89.72	C_13_H_28_	184.219	
	1002-43-3	Undecane, 3-methyl-	89.41	C_12_H_26_	170.203	
	53366-38-4	Cyclopentane, (2-methylbutyl)-	88.11	C_10_H_20_	140.157	
	17301-32-5	Undecane, 4,7-dimethyl-	85.37	C_13_H_28_	184.219	
	563-16-6	Hexane, 3,3-dimethyl-	82.51	C_8_H_18_	114.141	
Hydrocarbons/ alkenes	629-89-0	1-Octadecyne	88.42	C_18_H_34_	250.266	n/a Starter compounds for several additives and polymers or are formed as a by-product in the olefin polymerisation [64].
	765-13-9	1-Pentadecyne	87.09	C_15_H_28_	208.219	
	74685-30-6	5-Eicosene	82.09	C_20_H_40_	280.313	
Halogenated hydrocarbons	4292-19-7	Dodecane, 1-iodo-	90.5	C_12_H_25_I	296.1	n/a Toxicity of chlorinated hydrocarbons is documented, and these compounds have been considered as persistent organic pollutants [65,66].
	1000406-32-0	Tetracosane, 1-iodo-	85.6	C_24_H_49_I	464.288	
Cyclic hydrocarbons	294-62-2	Cyclododecane	90.1	C_12_H_24_	168.188	n/a Probably reaction products or decomposition products [66].
Plastic additives/UV stabilisers	1843-05-6	Octabenzone	94.19	C_21_H_26_O_3_	326.188	*Uvinul 3008* UV stabiliser used to protect polymers against damage by UV [67].
	5466-77-3	2-Propenoic acid, 3-(4-methoxyphenyl)-, 2-ethylhexyl ester	92.45	C_18_H_26_O_3_	290.188	n/a UV-B filter, a common ingredient in sunscreen and other skincare products to minimise DNA photodamage [68].
	97-76-4	2,4-Di-tert-butylphenol	98.0	C_14_H_22_O	206.167	n/a
	764-42-1	Fumaronitrile	95.1	C_4_H_2_N_2_	78.022	n/a
	2082-79-3	Benzenepropanoic acid, 3,5-bis(1,1-dimethylethyl)-4-hydroxy-, octadecyl ester	88.13	C_35_H_62_O_3_	530.47	*Irganox 1076*
Plastic additives/ phtalathes	84-69-5	1,2-Benzenedicarboxylic acid, bis(2-methylpropyl) ester	91.99	C_16_H_22_O_4_	278.152	*DiBP*
	117-81-7	Bis(2-ethylhexyl) phthalate	94.6	C_24_H_38_O_4_	390.277	*DEHP* reprotoxic category 2, T (toxic) [69].
	84-66-2	Diethyl phthalate	88.20	C_12_H_14_O_4_	222.089	*DEP* most broadly used in medicinal products [70].
	84-74-2	Dibutyl phthalate	87.10	C_16_H_22_O_4_	278.152	*DBP* reprotoxic category 3, T (toxic), N (dangerous for the environment) [71,72]; most widely used in medicinal products.
	28029-89-2	Didecan-2-yl phthalate	87.3	C_28_H_46_O_4_	446.34	*1,2-Benzenedicarboxylic acid*
Plastic additives/ antioxidants	3896-11-5	2-tert-Butyl-6-(5-chloro-2H-benzotriazol- 2-yl)-4-methylphenol	89.61	C_17_H_18_ClN_3_O	315.114	*Bumetrizole.* Used to slow the oxidation process of the polymers exposed to UV light [71,72].
	101-72-4	1,4-Benzenediamine, N-(1-methylethyl)-N’-phenyl-	86.59	C_15_H_18_N_2_	226.147	*Santoflex IPPD*
	95906-11-9	Tris(2,4-di-tert-butylphenyl) phosphate	86.12	C_42_H_63_O_4_P	662.446	n/a
	15721-78-5	Benzenamine, 4-(1,1,3,3-tetramethylbutyl)-N-[4-(1,1,3,3-tetramethylbutyl)phenyl]-	84.4	C_28_H_43_N	393.34	n/a
Intermediate	4337-65-9	Hexanedioic acid, mono(2-ethylhexyl)ester	91.05	C_14_H_26_O_4_	258.183	n/a
*Alcohols*	10042-59-8	1-Heptanol, 2-propyl-	95.90	C_10_H_22_O	158.167	Chemical intermediate or internal lubricant. Impurities or degradation products [73,74,75]. Screening of plastic toys and feeding utensils for babies showed the presence of alcohols [76,77]. Possible degradation products of plastic [26,63,78].
	3913-02-8	1-Octanol, 2-butyl-	86.88	C_12_H_26_O	186.198	
	54004-41-0	1-Pentanol, 4-methyl-2-propyl-	84.85	C_9_H_20_O	144.151	
Biofilm and algae compounds	83-47-6	Gamma-sitosterol	82.02	C_29_H_50_O	414.386	n/a
	201358-24-9	24-Noroleana-3,12-diene	88.59	C_29_H_46_	394.36	n/a
	502-69-2	2-Pentadecanone, 6,10,14-trimethyl-	88.43	C_18_H_36_O	268.277	n/a

## Supplementary Materials

The following are available online at https://www.mdpi.com/2305-6304/8/4/100/s1, Figure S1: GC–MS total ion chromatograms (TICs) of PAHs related to microplastic extract of the April 2017 campaign. Figure S2: GC–MS total ion chromatograms (TICs) of PAHs related to microplastic extract of the February 2017 campaign. Figure S3: GC–MS total ion chromatograms (TICs) of PAHs related to microplastic extract of the December 2017 campaign. Figure S4: GC–MS total ion chromatograms (TIC) of PAHs related to microplastic extract of the May 2018 campaign. Table S1: Lists of PCB congeners and their precursor and product ions used for the MS/MS method for GC–MS analysis. * Compound is an internal standard. Table S2: lists of OCPs and their precursor and product ions used for the MS/MS method for GC–MS analysis. Table S3: Lists of PAHs their precursor ions used for the MS/MS method for GC–MS analysis. Table S4:PCB recoveries calculated for each sample using [13C12]PCB 104 as an internal standard. Table S5: PAH and OCP recoveries calculated on spiked matrix blanks. Table S6: Limit of detection (LOD) values for the validated methods, in ng/g^−1^ plastic. Table S7: Concentrations of 16 PCBs congeners, expressed as ng/g, found on environmental microplastic samples (April 2017, February 2017, December 2017, May 2018), and on virgin pre-production microplastics (virgin PE, virgin colored PE, virgin PP). Virgin PE and PP were colorless pellets while Virgin colored PE was green microparticles (<500 µm). Congeners indicated in red are the dioxin-like ones. Table S8: Concentrations of 16 PCB congeners, expressed as ng/g, found on environmental microplastic samples (April 2017, February 2017, December 2017, May 2018), and on virgin pre-production microplastics (virgin PE, virgin colored PE, virgin PP). Virgin PE and PP were colorless pellets while virgin colored PE was green microparticles (<500 µm). Congeners indicated in red are the dioxin-like ones. Table S9: Concentrations of 16 EPA-PAHs, expressed as ng/g, found on environmental microplastic samples (April 2017, February 2017, December 2017, May 2018) and on virgin pre-production microplastics (virgin PE, virgin colored PE, virgin PP). Virgin PE and PP were colorless pellets while virgin colored PE was green microparticles (< 500 µm). Table S10: Concentrations of eight OCPs, expressed as ng/g, found on environmental microplastic samples (April 2017, February 2017, December 2017, May 2018) and on virgin pre-production microplastics (virgin PE, virgin colored PE, virgin PP). Virgin PE and PP were colorless pellets while virgin colored PE was green microparticles (<500 µm). Table S11: Overview of 248 different types of compounds hypothetically identified on environmental microplastic samples (April 2017, February 2017, December 2017, May 2018) by the general screening. The CAS no., NIST probability match factor %, molecular weight, and chemical formula of each compound are also given in the table. Table S12: Accurate mass measurements and elemental compositions of compounds found on microplastic and their product ions using HRGC-MS analysis. Table S13: Comparison of high-resolution spectra of 15 hypothetically identified compounds by spectra of NIST 17 library.
