# Supplementary material for: A Relevant Screening of Organic Contaminants Present on Freshwater and Pre-Production Microplastics
Source: Toxics. 2020 Nov 9;8(4):100. doi: 10.3390/toxics8040100 (PMC7712310; doi:10.3390/toxics8040100)
Supplement: Supplementary file 1 [file toxics-08-00100-s001.pdf]

Supplementary Materials: A Relevant Screening of Organic Contaminants Present on Freshwater and Pre-Production Microplastics

Claudia Campanale, Georg Dierkes, Carmine Massarelli, Giuseppe Bagnuolo and Vito Felice Uricchio [1]

1. Materials and Methods

1.1. PCBs

All congeners were separated under the following conditions: carrier gas, helium with a constant flow rate of 1.4 mL/min, initial injector temperature of 75 °C ramped to 280 °C (held 2 min) at 14.5 °C/s.

Two ions were monitored for each PCB homologous group using MS/MS acquisition mode (Table S1). The mass spectrometer was used in electronic impact (EI) mode (70 eV electron energy), with ion source and transfer line temperatures set to 260 °C and 250 °C, respectively. The oven temperature was initially set at 80 °C for 2 min, increased at first to 160 °C at a rate of 25 °C/min and held for 1 min, then increased to 210 °C at a rate of 4 °C/min and held for 10 min, further increased to 280 °C at a rate of 10 °C/min and held for 1 min, and finally increased to 310 °C at a rate of 30 °C/min and held for 1 min.

Table S1. Lists of PCB congeners and their precursor and product ions used for the MS/MS method for GC–MS analysis. * Compound is an internal standard.

PCBs	Precursor Ion (*m*/*z*) [Da]	Product Ion (*m*/*z*) [Da]
CB-18	258	186, 188
CB-28	258	186, 188
CB-52	292	220, 222
CB-44	292	220, 222
CB-95	326	254, 256
CB-101	326	254, 256
CB-99	326	254, 256
CB-81	292	220, 222
CB-77	292	220, 222
CB-110	326	254, 256
CB-151	360	290, 288
CB-123	326	254,256
CB-149	360	290, 288
CB-118	326	254,256
CB-114	326	254,256
CB-146	360	290, 288
CB-153	360	290, 288
CB-105	326	254,256
CB-138	360	290, 288
CB-126	326	254,256
CB-187	395	324, 326
CB-183	395	324, 326
CB-128	360	290, 288
CB-167	360	290, 288
CB-177	395	324, 326
CB-156	360	290, 288
CB-157	360	290, 288
CB-180	395	324, 326
CB-169	360	290, 288
CB-170	395	324, 326
CB-189	395	324, 326
CB-104*	338	266, 268

1.2. OCPs

Organochlorine pesticides (α-HexaChloroCycloHexane, β-HexaChloroCycloHexane, γ-HexaChloroCycloHexane, δ-HexaChloroCycloHexane, aldrin, p,p′-DDT, p,p′-DDD, p,p′-DDE) were analyzed using MS/MS acquisition mode monitoring 2 ions for each compound (Table S2) under the following mass spectrometer conditions: electron impact (EI) mode with a standard electron energy of 70 eV, transfer line at 250 °C, damping gas at 2 mL/min, and ion source at 250 °C. Helium (He) was employed as the carrier gas, with a constant flow rate of 1.4mL/ min. The initial injector temperature was 75 °C, raised to 280 °C (held 2 min).

The oven temperature was initially set at 75 °C for 2 min, increased at first to 150 °C at a rate of 20 °C/min and held for 2 min, then increased to 260 °C at a rate of 3 °C/min and held for 2 min, and finally increased to 300 °C at a rate of 20 °C/min and held for 1 min.

Table S2. Lists of OCPs and their precursor and product ions used for the MS/MS method for GC–MS analysis.

**OCPs**	**Precursor Ion (*m*/*z*) [Da]**	**Product Ion (*m*/*z*) [Da]**
α-BHC	218	181, 183
β-BHC	218	181, 183
γ-BHC	218	181, 183
δ-BHC	218	181, 183
Aldrin	293	257, 258
DDE	318	246, 248
DDD	235	165
DDT	235	165
Endrin	281	245, 243

1.3. PAHs

Selected ion monitoring (SIM) of the ions (Table S3) was used to detect 16 EPA-PAHs (acenaphthylene, acenaphthene, fluorene, phenanthrene, anthracene, fluoranthene, pyrene, benz(a)anthracene, chrysene, benzo(b)fluoranthene, benzo(k)fluoranthene, benzo(a)pyrene, indeno(123-cd)pyrene, dibenzo(ah)anthracene, and benzo-(ghi)perylene).

The initial injector temperature was 40 °C ramped to 280 °C (held 2 min) at 14.5 °C/s using helium as carrier gas at a constant flow of 1.4mL/min.

The mass spectrometer was used in electronic impact (EI) mode (70 eV electron energy) with ion source and transfer line temperatures of 250 °C and 270 °C, respectively.

The oven temperature was initially set at 80 °C for 2 min, increased at first to 160 °C at a rate of 25 °C/min and held for 1 min, then increased to 210 °C at a rate of 4 °C/min and held for 10 min, further increased to 280 °C at a rate of 10 °C/min and held for 1 min, and finally increased to 310 °C at a rate of 30 °C/min and held for 1 min.

Table S3. Lists of PAHs their precursor ions used for the MS/MS method for GC–MS analysis.

**PAHs**	**Precursor Ion (*m*/*z*) [Da]**
Naphthalene	128
Acenaphthalene	152
Acenaphthene	154

Fluorene	166
Phenanthrene	178
Anthracene	178
Fluoranthene	202
Pyrene	202
Benz(a)anthracene	228
Chrysene	228
Benzo(b)fluoranthene	252
Benzo(k)fluoranthene	252
Benzo(a)pyrene	252
Indeno(123-cd)pyrene	276
Dibenzo(ah)anthracene	278
Benzo-(ghi)perylene	276

1.4. Compounds Identification and Quantification

The signal-to-noise ratio was higher than 3 for each compound revealed in each sample, and greater than 10 for pollutants in calibration standards, in order to guarantee that the correct identification of the target compounds' GC retention times matched those of the standard compounds within ± 0.3 minutes. PAH and OCP quantization were achieved using an external standard approach that involved the comparison of instrument responses from the sample to the responses from target analytes of known concentration in the calibration standards.

Calibration curves obtained by least squares regression were derived from a minimum of 5 standards of varying concentration prepared by serial dilution of a stock solution with an appropriate solvent.

Two multi-level calibration curves were used to define 2 different working ranges for each compound, covering the concentrations 2.5–50/25-500 ng/g for PAHs and 0.2–10/10–00 ng/g for OCPs. Otherwise, an internal standards calibration approach was used for the quantification of PCBs. This involves a comparison of instrument responses from the target compounds in the sample to responses of other standards added to the sample or extract before injection. A known and constant amount of internal standard (carbon-13 PCB 104) was spiked to each samples before extraction, and the recovery of the labelled compound was calculated with the following formula:

$$\frac{\text{Concentration found } (\mu g/mL)}{\text{Concentration spiked } (\mu g/mL)} \times 100 = \text{Recovery } (\%) \quad (1)$$

To calculate the analysis results, we determined the ratio of the peak area for the analyte to that for the internal standard, and the concentration was calculated using the calibration function.

1.5. Quality Assurance and Quality Control for Pollutant Analysis

Regarding quality assurance of pollutant analysis, the present study incorporated a set of quality control samples analyzed in each batch of analysis to determine the performance of the analytical method, including the following blanks:

Solvent blanks—15 mL of hexane evaporated to incipient dryness and re-solubilized into 0.5 mL nonane in order to check the amount of the signal that is due to the solvents used for samples extraction.

Procedural blanks—reagents without sample, spiked with a known amount of internal standard [13C12]PCB 104 into an empty tube analyzed with the same method of samples to determine blank levels of analytes.

Blank matrices—virgin colorless polyethene (PE) pre-production pellets, virgin colored polyethene (PE) pre-production microparticles (green particles <500 μm), and virgin colorless polypropylene (PP) pre-production pellets (cleaned with deionized purified water and air-dried) spiked with an internal standard solution containing the labelled compounds [13C12]PCB 104.

OPR (ongoing precision and recovery) blanks—consisting of virgin pre-production pellets spiked with a known amount of labelled PCB standard and natives' PCBs, PAHs, and OCPs to simulate samples in order to check the performance of recovery of the GC–MS system.

Environmental blanks—river water samples collected during the May campaign in 2018 in order to compare the concentration of contaminants adsorbed on microplastics to the concentration of pollutants found in ambient river water.

Internal standard solution (using [13C12]PCB 104) was added before extraction of real samples, and PCB recoveries were calculated for each sample. The samples analyzed were found to fall in the range from 55% to 105% recovery (Table S4).

Table S4. PCB recoveries calculated for each sample using [13C12]PCB 104 as an internal standard.

Sample	Recovery [13C12]PCB 104 (%)
April 2017	70
February 2017	60
December 2017	55
May 2018	70
Virgin PE	105
Virgin colored PE	61
Virgin PP	101

Initial precision and recovery (IPR): Validation of both PAH and OCP extraction methods was performed, before determination of real samples, by analyzing virgin polyethene and polypropylene pellets spiked with a mid-range of native PAH and OCP standard in order to check the performance of recovery of the extraction.

The recoveries of the 16 PAHs and 8 pesticides in the matrix-spiked blanks were acceptable, consisting of a mean of 70% (Table S5).

PAH and OCP recoveries in real samples were assumed to be similar to that obtained for matrix-spiked blanks.

Table S5. PAH and OCP recoveries calculated on spiked matrix blanks.

PAHs	Recovery (%)	OCPs	Recovery (%)
Naphtalene	65	Alfa-BHC	70
Acenaphtylene	74	Beta-BHC	72
Acenaphtene	75	Delta-BHC	75
Fluorene	69	Gamma-BHC	69
Phenanthrene	66	Aldrin	80
Antracene	63	DDT	78
Fluoranthene	60	DDD	79
Pyrene	60	DDE	85
Benzo(a)antracene	62		
Chrysene	63		
Benzo(b)fluoranthene	71		
Benzo(k)fluoranthene	65		
Benzo(a)pyrene	61		
Indeno(123cd)pyrene	65		
Dibenzo(ah)anthracene	64		
Benzo(ghi)perylene	63		

The limits of detection (LODs) were set as the lower points of the calibration curves and ranged from 2.5 to 25 ng/g for PAHs, 0.2 to 10 ng/g for pesticides, and 0.2 ng/g for PCBs (Table S6).

The reported results in this study were corrected for the procedural blanks values. The solvent blank values and the environmental blanks were always below the detection limits.

Table S6. Limit of detection (LOD) values for the validated methods, in ng/g⁻¹ plastic.

PAHs	LOD ng/g^{-1}	OCPs	LOD ng/g^{-1}	PCBs	LOD ng/g^{-1}
Naphtalene	2.5	Alfa-BHC	1.0	CB-18	0.2
Acenaphtylene	5.0	Beta-BHC	1.0	CB-28	0.2
Acenaphtene	3.0	Gamma-BHC	1.0	CB-52	0.2
Fluorene	10.0	Delta-BHC	1.0	CB-44	0.2
Phenantrene	25.0	Aldrin	10	CB-95	0.2
Antracene	10.0	p-p′DDE	0.5	CB-101	0.2
Fluoranthene	10.0	p-p′DDD	0.2	CB-99	0.2
Pyrene	25.0	p-p′DDT	10	CB-81	0.2
Benzo(a)antracene	10.0			CB-77	0.2
Chrysene	25.0			CB-110	0.2
Benzo(b)fluoranthene	10.0			CB-151	0.2
Benzo(k)fluoranthene	25.0			CB-123	0.2
Benzo(a)pyrene	25.0			CB-149	0.2
Indeno(123cd)pyrene	25.0			CB-118	0.2
Dibenzo(ah)anthracene	25.0			CB-114	0.2
Benzo(ghi)perylene	25.0			CB-146	0.2
				CB-153	0.2
				CB-105	0.2
				CB-138	0.2
				CB-126	0.2
				CB-187	0.2
				CB-183	0.2
				CB-128	0.2
				CB-167	0.2
				CB-177	0.2
				CB-156	0.2
				CB-157	0.2
				CB-180	0.2
				CB-169	0.2
				CB-170	0.2
				CB-189	0.2

2. Results

2.1. PCBs

Table S7. Concentrations of 16 PCBs congeners, expressed as ng/g, found on environmental microplastic samples (April 2017, February 2017, December 2017, May 2018), and on virgin pre-production microplastics (virgin PE, virgin colored PE, virgin PP). Virgin PE and PP were colorless pellets while Virgin colored PE was green microparticles (<500 μm). Congeners indicated in red are the dioxin-like ones.

Congener	18	28	44	52	95	101	99	81	77	110	151	123	149	118	114	146
Unit of measurement	ng/g	ng/g	ng/g	ng/g	ng/g	ng/g	ng/g	ng/g	ng/g	ng/g	ng/g	ng/g	ng/g	ng/g	ng/g	ng/g
PP pellets	<LOD	<LOD	<LOD	<LOD	<LOD	<LOD	<LOD	<LOD	<LOD	<LOD	<LOD	<LOD	<LOD	<LOD	<LOD	<LOD

PE microplastics	<LOD	<LOD	<LOD	<LOD	<LOD	<LOD	<LOD	<LOD	<LOD	0.50	<LOD	<LOD	<LOD	<LOD	<LOD	<LOD
PE pellets	<LOD	<LOD	<LOD	<LOD	<LOD	<LOD	<LOD	<LOD	<LOD	<LOD	<LOD	<LOD	<LOD	<LOD	<LOD	<LOD
May 2018	<LOD	0.44	<LOD	3.33	0.73	1.32	0.67	<LOD	<LOD	0.92	0.03	0.02	1.06	0.61	<LOD	<LOD
Dec 2017	<LOD	0.74	<LOD	0.95	0.58	1.30	0.85	<LOD	<LOD	0.47	0.03	0.02	0.77	0.47	<LOD	<LOD
Apr 2017	<LOD	0.93	<LOD	0.5	0.75	1.17	0.57	<LOD	0.12	0.33	0.29	0.09	1.6	0.48	<LOD	<LOD
Feb 2017	<LOD	1.73	<LOD	1.37	1.27	1.64	0.74	<LOD	0.28	0.72	0.22	<LOD	1.19	0.4	<LOD	<LOD
Water samples	<LOD	<LOD	<LOD	<LOD	<LOD	<LOD	<LOD	<LOD	<LOD	<LOD	<LOD	<LOD	<LOD	<LOD	<LOD	<LOD

Table S8. Concentrations of 16 PCB congeners, expressed as ng/g, found on environmental microplastic samples (April 2017, February 2017, December 2017, May 2018), and on virgin pre-production microplastics (virgin PE, virgin colored PE, virgin PP). Virgin PE and PP were colorless pellets while virgin colored PE was green microparticles (<500µm). Congeners indicated in red are the dioxin-like ones.

**Congener**	**153**	**105**	**138**	**126**	**187**	**183**	**128**	**167**	**177**	**156**	**157**	**180**	**169**	**170**	**189**	**Total**
Unit of measurement	ng/g	ng/g	ng/g	ng/g	ng/g	ng/g	ng/g	ng/g	ng/g	ng/g	ng/g	ng/g	ng/g	ng/g	ng/g	ng/g
PP pellets	<LOD	<LOD	<LOD	<LOD	<LOD	<LOD	<LOD	<LOD	<LOD	<LOD	<LOD	<LOD	<LOD	<LOD	<LOD	<LOD
PE microplastics	<LOD	<LOD	<LOD	<LOD	<LOD	<LOD	<LOD	<LOD	<LOD	<LOD	<LOD	<LOD	<LOD	<LOD	<LOD	0.54
PE pellets	<LOD	<LOD	<LOD	<LOD	<LOD	<LOD	<LOD	<LOD	<LOD	<LOD	<LOD	<LOD	<LOD	<LOD	<LOD	<LOD
May 2018	2.0	<LOD	3.0	<LOD	<LOD	<LOD	<LOD	<LOD	<LOD	<LOD	<LOD	1.12	<LOD	<LOD	<LOD	15.26
Dec 2017	0.69	<LOD	1.01	<LOD	<LOD	<LOD	<LOD	<LOD	<LOD	<LOD	<LOD	<LOD	<LOD	<LOD	<LOD	7.87
Apr 2017	2.33	<LOD	3.1	<LOD	<LOD	<LOD	<LOD	<LOD	<LOD	<LOD	<LOD	1.48	<LOD	<LOD	<LOD	13.67
Feb 2017	1.59	<LOD	2.24	<LOD	<LOD	<LOD	<LOD	<LOD	<LOD	<LOD	<LOD	1.37	<LOD	<LOD	<LOD	14.75
Water samples	<LOD	<LOD	<LOD	<LOD	<LOD	<LOD	<LOD	<LOD	<LOD	<LOD	<LOD	<LOD	<LOD	<LOD	<LOD	<LOD

LOD: limit of detection.

2.2. PAHs

Table S9. Concentrations of 16 EPA-PAHs, expressed as ng/g, found on environmental microplastic samples (April 2017, February 2017, December 2017, May 2018) and on virgin pre-production microplastics (virgin PE, virgin colored PE, virgin PP). Virgin PE and PP were colorless pellets while virgin colored PE was green microparticles (< 500 µm).

**PAHs**	**Naph**	**Acen**	**Acen**	**Fluo**	**Phen**	**Antr**	**Fluo**	**Pyre**	**Benz**	**Chry**	**Benz**	**Benz**	**Benz**	**Inde**	**Dibe**	**Benz**	**TO**
Unit of measurement	ng/g	ng/g	ng/g	ng/g	ng/g	ng/g	ng/g	ng/g	ng/g	ng/g	ng/g	ng/g	ng/g	ng/g	ng/g	ng/g	ng/g
PE pellets	28.43	<LOD	<LOD	<LOD	<LOD	<LOD	<LOD	<LOD	<LOD	<LOD	<LOD	<LOD	<LOD	1.47	<LOD	<LOD	29.90
PE microplastics	<LOD	<LOD	2.72	<LOD	26.67	<LOD	25.84	17.63	<LOD	<LOD	<LOD	<LOD	<LOD	<LOD	<LOD	<LOD	72.85
PP pellets	14.90	<LOD	<LOD	29.11	<LOD	<LOD	17.48	4.8	<LOD	<LOD	<LOD	<LOD	<LOD	2.42	<LOD	<LOD	68.71
May 2018	<LOD	1.4	<LOD	<LOD	29.64	<LOD	32.37	26.99	<LOD	10.11	<LOD	<LOD	<LOD	5.18	<LOD	14.61	120.30
Dec 2017	<LOD	4.63	<LOD	33.70	46.00	<LOD	31.70	15.37	<LOD	3.33	<LOD	<LOD	<LOD	5.54	<LOD	19.79	160.06
Apr 2017	<LOD	15.95	11.70	29.74	56.59	<LOD	33.17	41.30	<LOD	7.30	<LOD	<LOD	<LOD	3.38	<LOD	15.95	215.07

Feb 2017	<LOD	9.72	6.61	32.60	77.69	<LOD	44.65	54.34	<LOD	10.39	<LOD	<LOD	<LOD	9.81	<LOD	23.30	269.12
Water samples	<LOD	<LOD	<LOD	<LOD	<LOD	<LOD	<LOD	<LOD	<LOD	<LOD	<LOD	<LOD	<LOD	<LOD	<LOD	<LOD	<LOD

LOD: limit of detection.

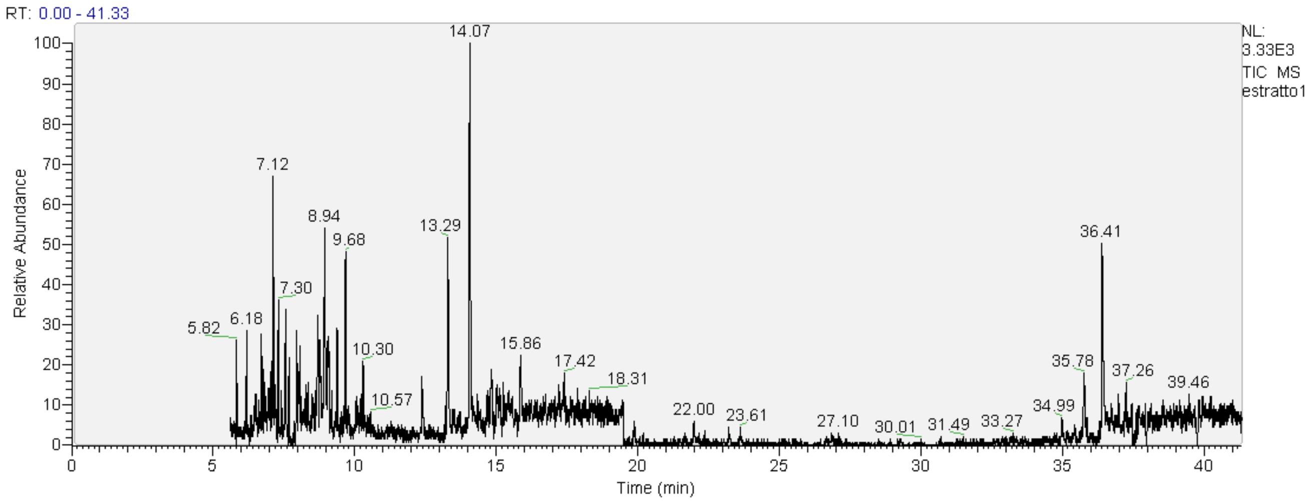

Figure S1. GC–MS total ion chromatograms (TICs) of PAHs related to microplastic extract of the April 2017 campaign.

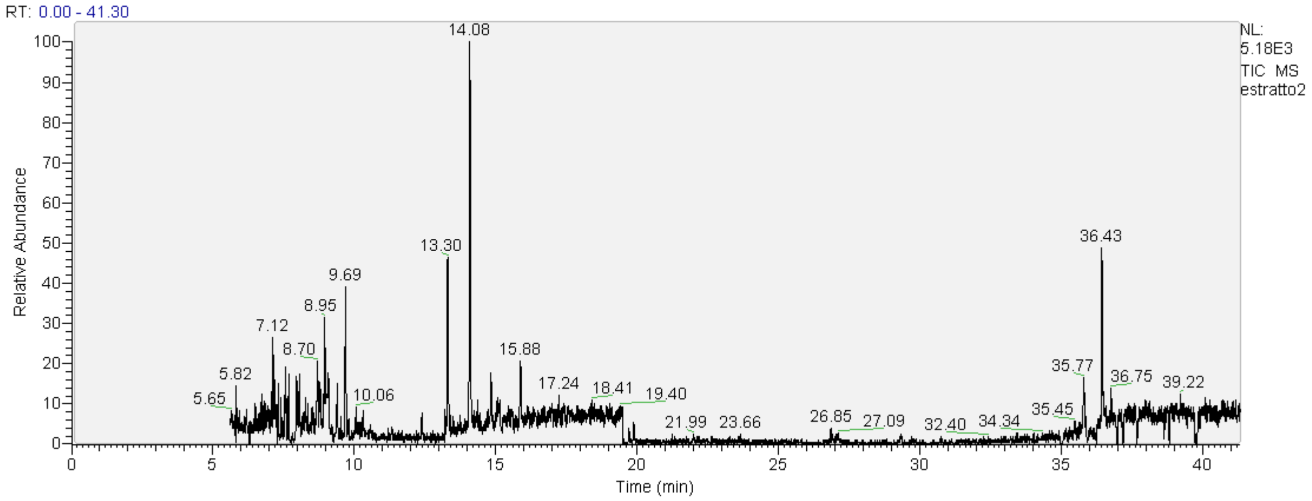

Figure S2. GC–MS total ion chromatograms (TICs) of PAHs related to microplastic extract of the February 2017 campaign.

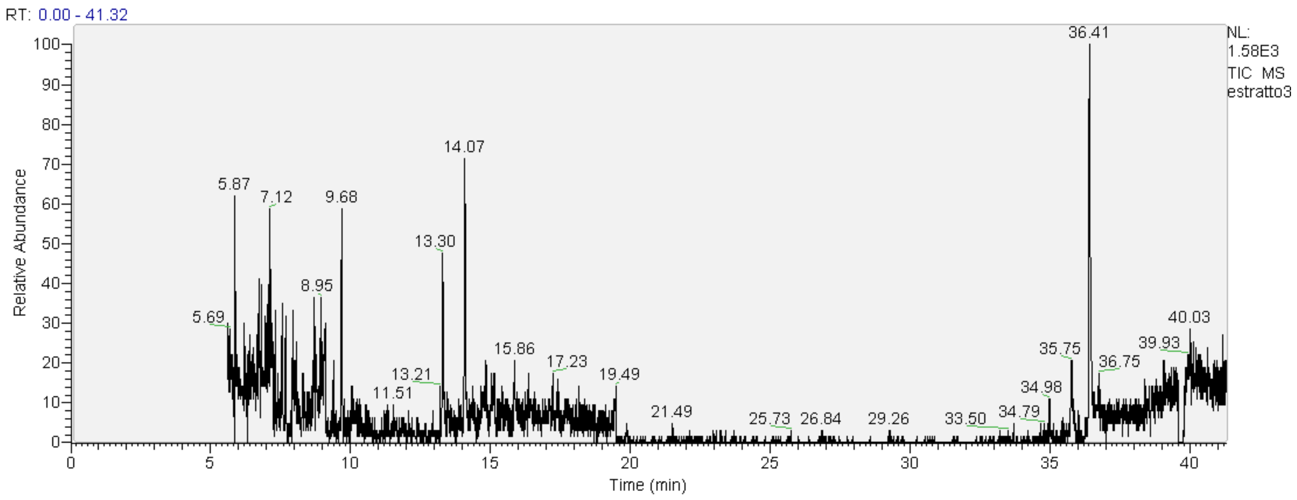

Figure S3. GC–MS total ion chromatograms (TICs) of PAHs related to microplastic extract of the December 2017 campaign.

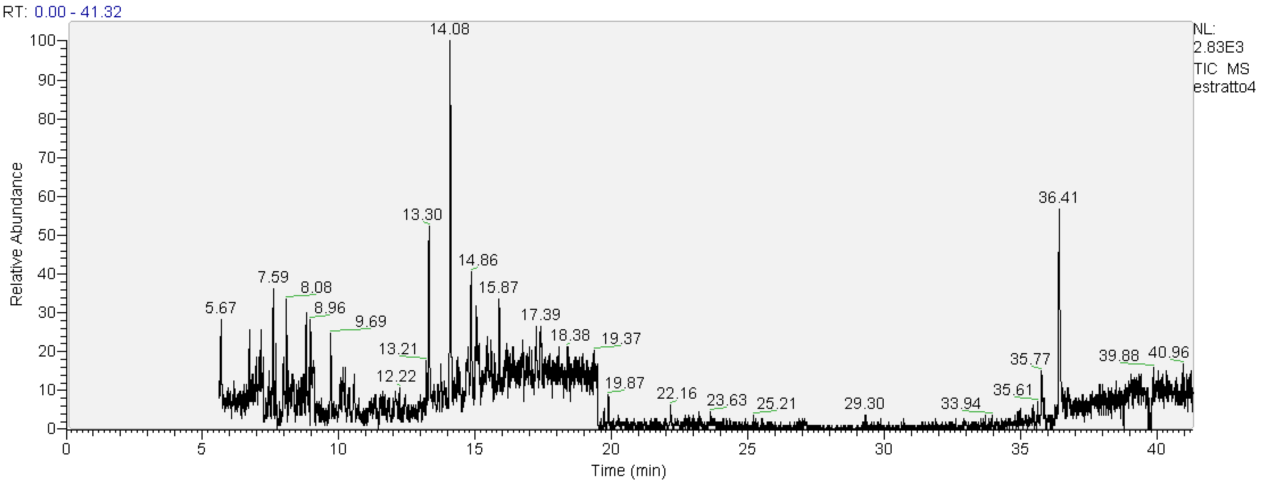

Figure S4. GC–MS total ion chromatograms (TIC) of PAHs related to microplastic extract of the May 2018 campaign.

2.3. OCPs

Table S10. Concentrations of eight OCPs, expressed as ng/g, found on environmental microplastic samples (April 2017, February 2017, December 2017, May 2018) and on virgin pre-production microplastics (virgin PE, virgin colored PE, virgin PP). Virgin PE and PP were colorless pellets while virgin colored PE was green microparticles (<500 µm).

OCPs	Alfa-HCH	Beta-HCH	Gamma-HCH	Delta-HCH	Aldrin	DDE	DDD	DDT	TOT
u.d.m.	ng/g	ng/g	ng/g	ng/g	ng/g	ng/g	ng/g	ng/g	ng/g
PE pellets	<LOD	<LOD	<LOD	<LOD	<LOD	<LOD	<LOD	<LOD	<LOD
PE microplastics	<LOD	<LOD	<LOD	<LOD	<LOD	<LOD	<LOD	<LOD	<LOD
PP pellets	<LOD	<LOD	<LOD	<LOD	<LOD	<LOD	<LOD	<LOD	<LOD
May 2018	<LOD	<LOD	<LOD	<LOD	<LOD	23.71	9.33	30.75	63.79
Dec 2017	<LOD	<LOD	<LOD	<LOD	<LOD	15.30	<LOD	31.53	46.84
Apr 2017	<LOD	<LOD	<LOD	<LOD	<LOD	14.53	<LOD	<LOD	14.53
Feb 2017	<LOD	<LOD	1.98	<LOD	<LOD	12.19	9.29	<LOD	23.46
Water samples	<LOD	<LOD	<LOD	<LOD	<LOD	<LOD	<LOD	<LOD	<LOD

LOD: limit of detection.

2.4. Non-Target Screening

Table S11. Overview of 248 different types of compounds hypothetically identified on environmental microplastic samples (April 2017, February 2017, December 2017, May 2018) by the general screening. The CAS no., NIST probability match factor %, molecular weight, and chemical formula of each compound are also given in the table.

Number Compound	Compound Name	Match Factor	Formula	CAS#	Library Molecular Weight
1	(2,3-Diphenylcyclopropyl)methyl phenyl sulfoxide, trans-	73.14	$C_{22}H_{20}OS$	131758-71-9	332.123
2	Gamma-sitosterol	91.83	$C_{29}H_{50}O$	83-47-6	414.386
3	1,19-Eicosadiene	71.05	$C_{20}H_{38}$	14811-95-1	278.297
4	1,2,2-Trimethylpropyl trifluoroacetate	77.3	$C_8H_{13}F_3O_2$	116465-21-5	198.087
5	1,2-Benzenedicarboxylic acid, diisodecyl ester	73.37	$C_{28}H_{46}O_4$	26761-40-0	446.34
6	1,2-Benzenedicarboxylic acid, bis(2-methylpropyl) ester	91.99	$C_{16}H_{22}O_4$	84-69-5	278.152
7	1,3-Cyclopentadiene	77.8	C_5H_6	542-92-7	66.047
8	1,4-Benzenediamine, N-(1-methylethyl)-N′-phenyl-	81.27	$C_{15}H_{18}N_2$	101-72-4	226.147
9	1,4-Cyclohexadiene	78.1	C_6H_8	628-41-1	80.063
10	1,7-Dimethyl-4-(1-methylethyl)cyclodecane	77.5	$C_{15}H_{30}$	645-10-3	210.235
11	1-[1,2,4]Triazol-1-ylethanone	72.1	$C_4H_5N_3O$	15625-88-4	111.043
12	10-Heneicosene, 11-phenyl-	70	$C_{27}H_{46}$	6703-78-2	370.36
13	17-(1,5-Dimethyl-3-phenylthiohex-4-enyl)-4,4,10,13,14-pentamethyl-2,3,4,5,6,7,10,11,12,13,14,15,16,17-tetradecahydro-1H-cyclopent(a)phenanthren-3-ol	74.43	$C_{36}H_{54}OS$	1000195-18-4	534.39
14	1-Decanol, 2-ethyl-	91.2	$C_{12}H_{26}O$	21078-65-9	186.198
15	1-Decanol, 2-hexyl-	81.56	$C_{16}H_{34}O$	2425-77-6	242.261
16	1-Decanol, 2-octyl-	94	$C_{18}H_{38}O$	45235-48-1	270.292
17	1-Docosanol, acetate	87.52	$C_{24}H_{48}O_2$	822-26-4	368.365
18	1-Dodecanol, 2-octyl-	92.2	$C_{20}H_{42}O$	5333-42-6	298.324
19	1-Eicosanol	83.85	$C_{20}H_{42}O$	629-96-9	298.324
20	1-Heptanol, 2-propyl-	94	$C_{10}H_{22}O$	10042-59-8	158.167
21	1-Hexen-3-one	70	$C_6H_{10}O$	1629-60-3	98.073
22	1H-Indene, 1,1-dimethyl-	74.8	$C_{11}H_{12}$	18636-55-0	144.094
23	1H-Indene, 1,3-dimethyl-	78.3	$C_{11}H_{12}$	2177-48-2	144.094
24	1H-Pyrazole, 4,5-dihydro-5-methyl-	77.2	$C_4H_8N_2$	1568-20-3	84.069
25	1H-Tetrazole	78.7	CH_2N_4	288-94-8	70.028
26	1-Iodo-2-methylundecane	81.76	$C_{12}H_{25}I$	73105-67-6	296.1
27	1-Methyl-1H-1,2,4-triazole	80.1	$C_3H_5N_3$	6086-21-1	83.048
28	1-Nonylcycloheptane	78	$C_{16}H_{32}$	1000371-47-7	224.25
29	1-Octadecanesulphonyl chloride	75.17	$C_{18}H_{37}ClO_2S$	1000342-70-4	352.22
30	1-Octanol, 2-butyl-	86.9	$C_{12}H_{26}O$	3913-02-8	186.198
31	1-Oxa-3,4-diazacyclopentadiene	74.5	$C_2H_2N_2O$	288-99-3	70.017
32	1-Pentanone, 1-(4-methylphenyl)-	72.8	$C_{12}H_{16}O$	1671-77-8	176.12
33	1-Pentene, 3,4-dimethyl-	78.4	C_7H_{14}	7385-78-6	98.11
34	1-Pentene, 3-methyl-	71.9	C_6H_{12}	760-20-3	84.094
35	1-Propanol, dl-2-benzylamino-,	79.5	$C_{10}H_{15}NO$	6940-81-4	165.115
36	1-Tetradecene	80	$C_{14}H_{28}$	1120-36-1	196.219
37	1-Undecene, 11-chloro-	70.21	$C_{11}H_{21}Cl$	872-17-3	188.133
38	2,15-Hexadecanedione	73.18	$C_{16}H_{30}O_2$	18650-13-0	254.225
39	2,2,4,4,5,5,7,7-Octamethyloctane	70	$C_{16}H_{34}$	5171-85-7	226.266
40	2,2,6,6-Tetramethylheptane	75.6	$C_{11}H_{24}$	40117-45-1	156.188
41	2,2-Dimethylpropanoic anhydride	78.6	$C_{10}H_{18}O_3$	1538-75-6	186.126
42	2,3-Dimethyl-1-hexene	78.7	C_8H_{16}	16746-86-4	112.125
43	2,4,5-Trihydroxypyrimidine	81.2	$C_4H_4N_2O_3$	496-76-4	128.022
44	2,4-Di-tert-butylphenol	98	$C_{14}H_{22}O$	96-76-4	206.167
45	2,5-Cyclohexadiene-1,4-dione, 2,6-bis(1,1-dimethylethyl)-	75.4	$C_{14}H_{20}O_2$	719-22-2	220.146
46	2,6-Diisopropylnaphthalene	73.8	$C_{16}H_{20}$	24157-81-1	212.157
47	2-Bromotetradecane	77.69	$C_{14}H_{29}Br$	74036-95-6	276.145
48	2-Butene, 1,4-dibromo-, (E)-	71.7	$C_4H_6Br_2$	821-06-7	211.884

49	2-Difluoroamino-3-(N-fluoroimino)-1,1,1,2,4,4,4-heptafluoro- butene	77	$C_4F_{10}N_2$	13016-00-7	265.99
50	2-Heptene, 4-methyl-, (E)-	83.9	C_8H_{16}	66225-17-0	112.125
51	2-Hexanone, 3-methyl-4-methylene-	71.5	$C_8H_{14}O$	20690-71-5	126.104
52	2-Methylhexacosane	92.2	$C_{27}H_{56}$	1561-02-0	380.438
53	2-Octene	91.1	C_8H_{16}	111-67-1	112.125
54	2-Octene, (Z)-	90.1	C_8H_{16}	08/04/7642	112.125
55	2-Pentene, 5-bromo-2,3-dimethyl-	72.9	$C_7H_{13}Br$	56312-52-8	176.02
56	2-Piperidinone, N-[4-bromo-n-butyl]-	81.05	$C_9H_{16}BrNO$	195194-80-0	233.042
57	2-Propenoic acid, 3-(4-methoxyphenyl)-, 2-ethylhexyl ester	92.45	$C_{18}H_{26}O_3$	5466-77-3	290.188
58	3,4-Dihydroxyphenylglycol, 4TMS derivative	75.1	$C_{20}H_{42}O_4Si_4$	56114-62-6	458.216
59	3,4-Hexanedione	74.3	$C_6H_{10}O_2$	4437-51-8	114.068
60	3,5-di-tert-Butyl-4-hydroxybenzaldehyde	71.28	$C_{15}H_{22}O_2$	1620-98-0	234.162
61	3-Buten-2-ol	79	C_4H_8O	598-32-3	72.058
62	3-Heptene, 4-methyl-	87.3	C_8H_{16}	4485-16-9	112.125
63	3-Octene, (E)-	89.3	C_8H_{16}	14919-01-8	112.125
64	3-Octyne-2,5-dione, 6,6,7-trimethyl-	73.6	$C_{11}H_{16}O_2$	63922-61-2	180.115
65	4,4,6a,6b,8a,11,11,14b-Octamethyl-1,4,4a,5,6,6a,6b,7,8,8a,9,10,11,12,12a,14,14a,14b-octadecahydro-2H-picen-3-one	81.74	$C_{30}H_{48}O$	1000194-62-4	424.371
66	4H-Cyclopropa[5 ′,6′]benz[1′,2′:7,8]azuleno[5–b]oxiren-4-one, 8,8a-bis(acetyloxy)-2a-[(acetyloxy)methyl] 1,1a,1b,1c,2a,3,3a,6a,6b,7,8,8a-dodecahydro-6b-hydroxy-3a-methoxy-1,1,5,7-tetramethyl-, [1aR(1a.alpha.,1b.beta.,1c.alpha.,2a.alpha.,3a.alpha.,6a.alpha.,6b.alpha.,7.alpha.,8.beta.,8a.alpha.)]-	60.11	$C_{27}H_{36}O_{10}$	64869-55-2	520.231
67	4-Methyl-1,6-heptadien-4-ol	70.5	$C_8H_{14}O$	25201-40-5	126.104
68	4-Methyl-2,4-bis(p-hydroxyphenyl)pent-1-ene, 2TMS derivative	74.6	$C_{24}H_{36}O_2Si_2$	1000283-56-8	412.225
69	4′-Propoxy-2-methylpropiophenone	77.4	$C_{13}H_{18}O_2$	64436-60-8	206.131
70	5-(7a-Isopropenyl-4,5-dimethyl-octahydroinden-4-yl)-3-methyl-pent-2-en-1-ol	72.33	$C_{20}H_{34}O$	1000193-54-0	290.261
71	5-Eicosene, (E)-	82.09	$C_{20}H_{40}$	74685-30-6	280.313
72	5-Eicosyne	84.14	$C_{20}H_{38}$	74685-31-7	278.297
73	5-Isoxazolecarboxylic acid, 4,5-dihydro-5-methyl-, methyl ester, (R)-	75.5	$C_6H_9NO_3$	64018-42-4	143.058
74	5-Nonadecen-1-ol	85.84	$C_{19}H_{38}O$	1000131-11-9	282.292
75	7,9-Di-tertbutyl-1-oxaspiro[4,5]deca-6,9-dien-8-one	70.39	$C_{17}H_{26}O_2$	138345-00-3	262.193
76	7-Acetyl-6-ethyl-1,1,4,4-tetramethyltetralin	93.1	$C_{18}H_{26}O$	88-29-9	258.198
77	7-Heptadecene, 1-chloro-	70.46	$C_{17}H_{33}Cl$	56554-78-0	272.227
78	7-Hexadecene, (Z)-	77.3	$C_{16}H_{32}$	35507-09-6	224.25
79	9-Eicosyne	85.82	$C_{20}H_{38}$	71899-38-2	278.297
80	9-Tricosene, (Z)-	78.64	$C_{23}H_{46}$	27519-02-4	322.36
81	9-Undecenol, 2,10-dimethyl-	78.35	$C_{13}H_{26}O$	1000131-86-0	198.198
82	Acetone	84.6	C_3H_6O	67-64-1	58.042
83	Acenaphthene	82.13	$C_{12}H_{10}$	83-32-9	154.078
84	Acetyl valeryl	90.7	$C_7H_{12}O_2$	96-04-8	128.084
85	Anthracen-9-one, 10-heptyl-10-hydroxy-	70.2	$C_{21}H_{24}O_2$	1000157-53-8	308.178
86	Azetidine, 1,2-dimethyl-	75.9	$C_5H_{11}N$	51764-32-0	85.089
87	Behenic alcohol	73.6	$C_{22}H_{46}O$	661-19-8	326.355
88	Benzaldehyde, 4-(phenylmethoxy)-	73.9	$C_{14}H_{12}O_2$	4397-53-9	212.084
89	Benzenamine, 4-(1,1,3,3-tetramethylbutyl)-N-[4-(1,1,3,3-tetramethylbutyl)phenyl]-	84.4	$C_{28}H_{43}N$	15721-78-5	393.34
90	Benzene, (1-methyldodecyl)-	81.7	$C_{19}H_{32}$	4534-53-6	260.25
91	Benzene, (1-methylundecyl)-	75.7	$C_{18}H_{30}$	2719-61-1	246.235
92	Benzene, 1-ethynyl-4-methyl-	72.4	C_9H_8	766-97-2	116.063
93	Benzenepropanenitrile, beta.-oxo-	75.2	C_9H_7NO	614-16-4	145.053
94	Benzenepropanoic acid, 3,5-bis(1,1-dimethylethyl)-4-hydroxy-, octadecyl ester	88.13	$C_{35}H_{62}O_3$	2082-79-3	530.47
95	Benzenepropanol, alpha.-methyl-, acetate	75.6	$C_{12}H_{16}O_2$	10415-88-0	192.115
96	Benzocycloheptatriene	77.1	$C_{11}H_{10}$	264-09-5	142.078

97	Benzoic acid, 2,4,6-trimethyl-, 2,4,6-trimethylphenyl ester	70.23	$C_{19}H_{22}O_2$	1504-38-7	282.162
98	Benzoic acid, nonadecyl ester	71.76	$C_{26}H_{44}O_2$	1000340-23-2	388.334
99	Benzophenone	72.2	$C_{13}H_{10}O$	119-61-9	182.073
100	Bicyclo[3.1.1]heptan-3-one, 2,6,6-trimethyl-	82.8	$C_{10}H_{16}O$	18358-53-7	152.12
101	Bicyclo[3.1.1]heptan-3-one, 2,6,6-trimethyl-, (1.alpha.,2.alpha.,5.alpha.)-	80.6	$C_{10}H_{16}O$	547-60-4	152.12
102	Bis(2-ethylhexyl) phthalate	94.6	$C_{24}H_{38}O_4$	117-81-7	390.277
103	Bumetrizole	89.61	$C_{17}H_{18}ClN_3O$	729335	315.114
104	Butane, 2,2-dimethyl-	86	C_6H_{14}	75-83-2	86.11
105	Carbonic acid, decyl undecyl ester	73.1	$C_{22}H_{44}O_3$	1000383-16-0	356.329
106	Carbonic acid, dodecyl vinyl ester	86.9	$C_{15}H_{28}O_3$	1000382-54-8	256.204
107	Carbonic acid, eicosyl vinyl ester	81.4	$C_{23}H_{44}O_3$	1000382-54-3	368.329
108	Carbonic acid, octadecyl vinyl ester	73.2	$C_{21}H_{40}O_3$	1000382-54-4	340.298
109	Carbonic acid, tetradecyl vinyl ester	91.8	$C_{17}H_{32}O_3$	1000382-54-5	284.235
110	Chloroacetic acid, tetradecyl ester	73.1	$C_{16}H_{31}ClO_2$	18277-86-6	290.201
111	Chloromethane	78.5	CH_3Cl	74-87-3	49.992
112	Cholesterol	74.44	$C_{27}H_{46}O$	57-88-5	386.355
113	cis-2,4-Dimethylthiane, S,S-dioxide	73.9	$C_7H_{14}O_2S$	1000215-67-5	162.071
114	Cyclobutane, methyl-	82.4	C_5H_{10}	598-61-8	70.078
115	Cyclododecane	90.1	$C_{12}H_{24}$	294-62-2	168.188
116	Cycloheptasiloxane, tetradecamethyl-	83.6	$C_{14}H_{42}O_7Si_7$	107-50-6	518.132
117	Cyclooctane, methyl-	74.2	C_9H_{18}	1502-38-1	126.141
118	Cyclooctasiloxane, hexadecamethyl-	82.1	$C_{16}H_{48}O_8Si_8$	556-68-3	592.15
119	Cyclopenta[g]-2-benzopyran, 1,3,4,6,7,8-hexahydro-4,6,6,7,8,8-hexamethyl-	71.2	$C_{18}H_{26}O$	1222-05-5	258.198
120	Cyclopentane, (2-methylbutyl)-	88.11	$C_{10}H_{20}$	53366-38-4	140.157
121	Cyclopentane, 1,1,3-trimethyl-	71	C_8H_{16}	4516-69-2	112.125
122	Cyclopentane, 1-hexyl-3-methyl-	70.8	$C_{12}H_{24}$	61142-68-5	168.188
123	Cyclopentane, 1-pentyl-2-propyl-	76.5	$C_{13}H_{26}$	62199-51-3	182.203
124	Cyclopentane, 2-isopropyl-1,3-dimethyl-	78.4	$C_{10}H_{20}$	32281-85-9	140.157
125	Cyclopentane, butyl-	84.2	C_9H_{18}	2040-95-1	126.141
126	Cyclopentasiloxane, decamethyl-	92.2	$C_{10}H_{30}O_5Si_5$	541-02-6	370.094
127	Cyclopentene	85.2	C_5H_8	142-29-0	68.063
128	Cyclopentene, 1-methyl-	73	C_6H_{10}	693-89-0	82.078
129	Cyclopentene, 3-methyl-	72	C_6H_{10}	1120-62-3	82.078
130	Cyclopropene, 3-methyl-3-vinyl-	83.8	C_6H_8	71153-30-5	80.063
131	Cyclotetradecane	88.9	$C_{14}H_{28}$	295-17-0	196.219
132	Decane	84.59	$C_{10}H_{22}$	124-18-5	142.172
133	Decane, 1-iodo-	88.4	$C_{10}H_{21}I$	2050-77-3	268.069
134	Decane, 2,3,4-trimethyl-	81.3	$C_{13}H_{28}$	62238-15-7	184.219
135	Decane, 2,5,6-trimethyl-	89.72	$C_{13}H_{28}$	62108-23-0	184.219
136	Decane, 2,4-dimethyl-	90.7	$C_{12}H_{26}$	2801-84-5	170.203
137	Decane, 2,9-dimethyl-	86.1	$C_{12}H_{26}$	1002-17-1	170.203
138	Decane, 3,8-dimethyl-	86.2	$C_{12}H_{26}$	17312-55-9	170.203
139	Dibutyl phthalate	87.10	$C_{16}H_{22}O_4$	84-74-2	278.152
140	Dichloroacetic acid, nonyl ester	73.5	$C_{11}H_{20}Cl_2O_2$	83004-99-3	254.084
141	Dicyclohexyl phthalate	72.6	$C_{20}H_{26}O_4$	84-61-7	330.183
142	Didecan-2-yl phthalate	87.3	$C_{28}H_{46}O_4$	28029-89-2	446.34
143	Didecyl phthalate	76.6	$C_{28}H_{46}O_4$	84-77-5	446.34
144	Diethyl phthalate	88.20	$C_{12}H_{14}O_4$	84-66-2	222.089
145	Dimethylamine	73.6	C_2H_7N	124-40-3	45.058
146	Diphenyl sulfone	76.4	$C_{12}H_{10}O_2S$	127-63-9	218.04
147	Disparlure	77.9	$C_{19}H_{38}O$	29804-22-6	282.292
148	Disulfide, di-tert-dodecyl	75.51	$C_{24}H_{50}S_2$	27458-90-8	402.335
149	Docosane, 1-iodo-	85.5	$C_{22}H_{45}I$	1000406-31-9	436.257
150	Dodecane, 1-iodo-	90.5	$C_{12}H_{25}I$	4292-19-7	296.1
151	Dodecane, 2,6,11-trimethyl-	89.6	$C_{15}H_{32}$	31295-56-4	212.25
152	Dodecane, 2,7,10-trimethyl-	82.1	$C_{15}H_{32}$	74645-98-0	212.25
153	Dodecane, 5,8-diethyl-	71.09	$C_{16}H_{34}$	24251-86-3	226.266
154	Dotriacontane, 1-iodo-	75.5	$C_{32}H_{65}I$	1000406-32-4	576.413
155	E-14-Hexadecenal	76.64	$C_{16}H_{30}O$	330207-53-9	238.23
156	E-15-Heptadecenal	72.02	$C_{17}H_{32}O$	1000130-97-9	252.245
157	Eicosane, 1-iodo-	89.1	$C_{20}H_{41}I$	1000406-31-8	408.225
158	Eicosane, 2-methyl-	94.5	$C_{21}H_{44}$	1560-84-5	296.344

159	Eicosane, 7-hexyl-	94	$C_{26}H_{54}$	55333-99-8	366.423
160	Eicosane, 9-cyclohexyl-	73.1	$C_{26}H_{52}$	4443-61-2	364.407
161	Eicosyl octyl ether	83.1	$C_{28}H_{58}O$	1000406-38-8	410.449
162	Ethaneperoxoic acid, 1-cyano-1-[2-(2-phenyl-1,3-dioxolan-2-yl)ethyl]pentyl ester	82.3	$C_{19}H_{25}NO_5$	58422-92-7	347.173
163	Ethanethioic acid, S-(2-methylbutyl) ester	71.2	$C_7H_{14}OS$	69078-80-4	146.077
164	Ethanone, 1,1′-(1,3-phenylene)bis-	89.3	$C_{10}H_{10}O_2$	6781-42-6	162.068
165	Ethanone, 1-[4-(1-hydroxy-1-methylethyl)phenyl]-	84.6	$C_{11}H_{14}O_2$	54549-72-3	178.099
166	Ethyl 3-furoate	71.3	$C_7H_8O_3$	614-98-2	140.047
167	Ethylbenzene	84.3	C_8H_{10}	100-41-4	106.078
168	Fumaronitrile	95.1	$C_4H_2N_2$	764-42-1	78.022
169	Hentriacontane	93.29	$C_{31}H_{64}$	630-04-6	436.501
170	Heptadecane, 2,6-dimethyl-	85.3	$C_{19}H_{40}$	54105-67-8	268.313
171	Heptane, 1-chloro-	78.7	$C_7H_{15}Cl$	629-06-1	134.086
172	Heptane, 4-azido-	82.5	$C_7H_{15}N_3$	27126-22-3	141.127
173	Heptacosane	94.68	$C_{27}H_{56}$	593-49-7	380.438
174	Hexacosane, 1-iodo-	89.4	$C_{26}H_{53}I$	1000406-32-1	492.319
175	Hexadecane	96.33	$C_{16}H_{34}$	544-76-3	226.266
176	Hexadecane, 1-chloro-	68.01	$C_{16}H_{33}Cl$	4860-03-1	260.227
177	Hexadecane, 3-methyl-	89	$C_{17}H_{36}$	6418-43-5	240.282
178	Hexadecane, 5-butyl-	75.56	$C_{20}H_{42}$	6912-07-8	282.329
179	Hexane, 1-chloro-5-methyl-	87.4	$C_7H_{15}Cl$	33240-56-1	134.086
180	Hexane, 2,2,5,5-tetramethyl-	71.5	$C_{10}H_{22}$	1071-81-4	142.172
181	Hexane, 2,4-dimethyl-	88.86	C_8H_{18}	589-43-5	114.141
182	Hexane, 3,3-dimethyl-	82.51	C_8H_{18}	563-16-6	114.141
183	Hexane, 3-methyl-	91	C_7H_{16}	589-34-4	100.125
184	Indane	74.9	C_9H_{10}	496-11-7	118.078
185	Iodoacetylene	70.2	C_2HI	1000298-80-5	151.912
186	Limonen-6-ol, pivalate	74.01	$C_{15}H_{24}O_2$	1000124-59-2	236.178
187	Methanesulfonic acid, 7,8,9,10-tetrahydrocyclohepta[de]naphthalen-8-yl ester	71.4	$C_{15}H_{16}O_3S$	1000189-97-5	276.082
188	Naphthalene, 2-methyl-	81.77	$C_{11}H_{10}$	91-57-6	142.078
189	Nonadecane	84.34	$C_{19}H_{40}$	629-92-5	268.313
190	Nonane, 3-methylene-	84.1	$C_{10}H_{20}$	51655-64-2	140.157
191	Nonane, 5-methyl-5-propyl-	75.5	$C_{13}H_{28}$	17312-75-3	184.219
192	Octabenzone	94.19	$C_{21}H_{26}O_3$	1843-05-6	326.188
193	Octacosane	91.34	$C_{28}H_{58}$	394.454	630-02-4
194	Octacosane, 1-iodo-	76.8	$C_{28}H_{57}I$	1000406-32-2	520.351
195	Octacosane, 2-methyl-	76.9	$C_{29}H_{60}$	1560-98-1	408.47
196	Octadecane, 1-chloro-	75.47	$C_{18}H_{37}Cl$	3386-33-2	288.258
197	Octadecane, 1-iodo-	89	$C_{18}H_{37}I$	629-93-6	380.194
198	Octadecane, 2-methyl-	87.7	$C_{19}H_{40}$	1560-88-9	268.313
199	Octane	81.3	C_8H_{18}	111-65-9	114.141
200	Octane, 1,1′-oxybis-	81.66	$C_{16}H_{34}O$	629-82-3	242.261
201	Octane, 1-chloro-	88.5	$C_8H_{17}Cl$	111-85-3	148.102
202	Octane, 3-ethyl-2,7-dimethyl-	90.6	$C_{12}H_{26}$	62183-55-5	170.203
203	Octane, 3-methyl-6-methylene-	80.1	$C_{10}H_{20}$	74630-07-2	140.157
204	Octatriacontyl pentafluoropropionate	70.98	$C_{41}H_{77}F_5O_2$	1000351-89-1	696.584
205	Oxalic acid, allyl hexadecyl ester	91.4	$C_{21}H_{38}O_4$	1000309-24-4	354.277
206	Oxalic acid, allyl octadecyl ester	84.5	$C_{23}H_{42}O_4$	1000309-24-5	382.308
207	Oxalic acid, allyl pentadecyl ester	86.7	$C_{20}H_{36}O_4$	1000309-24-3	340.261
208	Oxalic acid, cyclobutyl octadecyl ester	79.3	$C_{24}H_{44}O_4$	1000309-70-8	396.324
209	Oxalic acid, isobutyl nonyl ester	80.86	$C_{15}H_{28}O_4$	1000309-37-4	272.199
210	Oxetane, 3-(1-methylethyl)-	76.5	$C_6H_{12}O$	10317-17-6	100.089
211	Pentadecane, 2,6,10-trimethyl-	88.4	$C_{18}H_{38}$	3892-00-0	254.297
212	Pentane, 2,3,3-trimethyl-	91.9	C_8H_{18}	560-21-4	114.141
213	Pentane, 2,2,3,4-tetramethyl-	92.25	C_9H_{20}	1186-53-4	128.157
214	Pentane, 3,3-dimethyl-	70.5	C_7H_{16}	562-49-2	100.125
215	Phenol, 2-(5-chloro-2H-benzotriazol-2-yl)-4,6-bis(1,1-dimethylethyl)-	79.28	$C_{20}H_{24}ClN_3O$	3864-99-1	357.161
216	Phthalic acid, heptyl tridec-2-yn-1-yl ester	82.05	$C_{28}H_{42}O_4$	1000315-44-0	442.308
217	Phthalic acid, isobutyl tridec-2-yn-1-yl ester	75.22	$C_{25}H_{36}O_4$	1000315-44-3	400.261
218	Phthalic acid, nonyl tridec-2-yn-1-yl ester	71.83	$C_{30}H_{46}O_4$	1000315-44-2	470.34

219	Phthalic acid, pentyl tridec-2-yn-1-yl ester	76.1	$C_{26}H_{38}O_4$	1000315-43-8	414.277
220	Propanal, 2-propenylhydrazone	70.8	$C_6H_{12}N_2$	19031-78-8	112.1
221	Propane, 2-isocyanato-	74.3	C_4H_7NO	1795-48-8	85.053
222	Propane, 2-methyl-1-nitro-	89.4	$C_4H_9NO_2$	625-74-1	103.063
223	Propargyl alcohol	94.1	C_3H_4O	107-19-7	56.026
224	Propyl pyruvate	79.6	$C_6H_{10}O_3$	1000431-41-8	130.063
225	Pyrolo[3,2-d]pyrimidin-2,4(1H,3H)-dione	88.8	$C_6H_5N_3O_2$	65996-50-1	151.038
226	Silane, diethylheptyloxyoctadecyloxy-	70.9	$C_{29}H_{62}O_2Si$	1000363-96-0	470.452
227	Silane, dimethyl(docosyloxy)butoxy-	64.71	$C_{28}H_{60}O_2Si$	1000347-86-1	456.436
228	Silicon tetrafluoride	70.7	F_4Si	7783-61-1	103.971
229	Succinic acid, 2-chloro-6-fluorophenyl 4-methoxybenzyl ester	78.7	$C_{18}H_{16}ClFO_5$	1000389-69-2	366.067
230	Sulfurous acid, butyl cyclohexylmethyl ester	70.3	$C_{11}H_{22}O_3S$	1000309-21-4	234.129
231	Sulfurous acid, butyl dodecyl ester	80	$C_{16}H_{34}O_3S$	1000309-17-9	306.223
232	Sulfurous acid, butyl heptadecyl ester	78.99	$C_{21}H_{44}O_3S$	1000309-18-4	376.301
233	Sulfurous acid, hexyl pentadecyl ester	83.91	$C_{21}H_{44}O_3S$	1000309-13-7	376.301
234	Sulfurous acid, pentadecyl 2-propyl ester	77.57	$C_{18}H_{38}O_3S$	1000309-12-6	334.254
235	Tetracosane, 11-decyl-	92.3	$C_{34}H_{70}$	55429-84-0	478.548
236	Tetracosane, 1-iodo-	85.6	$C_{24}H_{49}I$	1000406-32-0	464.288
237	Tetradecane, 1-iodo-	83.5	$C_{14}H_{29}I$	19218-94-1	324.131
238	Toluene	92.9	C_7H_8	108-88-3	92.063
239	tri(2-Ethylhexyl) trimellitate	77.3	$C_{33}H_{54}O_6$	3319-31-1	546.392
240	Triacontane, 1-iodo-	88.6	$C_{30}H_{61}I$	1000406-32-3	548.382
241	Tridecane	91.79	$C_{13}H_{28}$	629-50-5	184.219
242	Tris(2,4-di-tert-butylphenyl) phosphate	76.4	$C_{42}H_{63}O_4P$	95906-11-9	662.446
243	Undecane, 3,7-dimethyl-	84.3	$C_{13}H_{28}$	17301-29-0	184.219
244	Undecane, 4,7-dimethyl-	85.37	$C_{13}H_{28}$	17301-32-5	184.219
245	Undecane, 3,8-dimethyl-	81.4	$C_{13}H_{28}$	17301-30-3	184.219
246	Undecane, 3-methyl-	89.41	$C_{12}H_{26}$	1002-43-3	170.203
247	Valeric anhydride	75.1	$C_{10}H_{18}O_3$	2082-59-9	186.126
248	Vinyl 10-undecenoate	74.8	$C_{13}H_{22}O_2$	5299-57-0	210.162

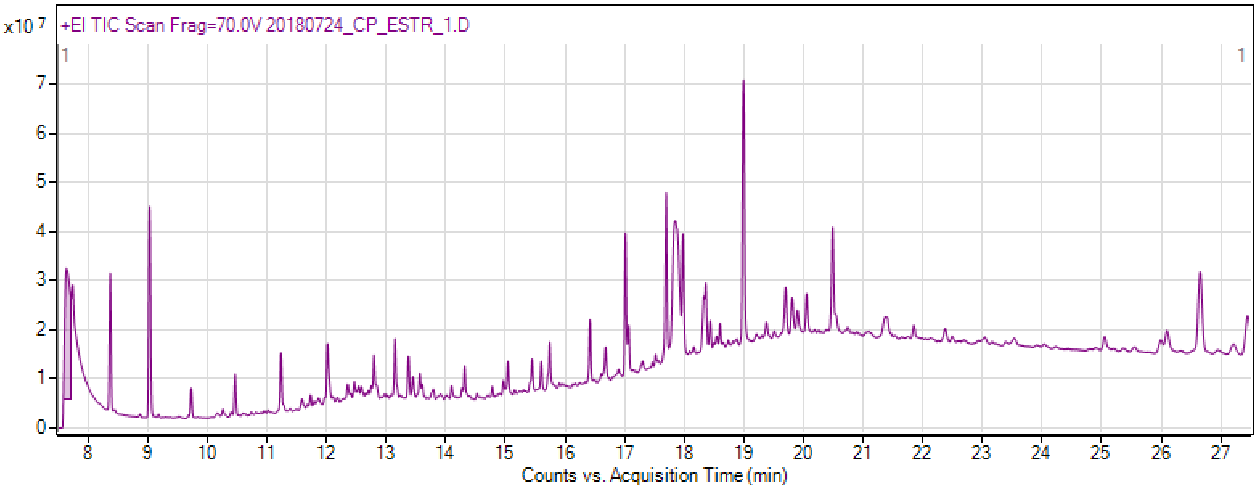

Figure S5. HRGC–MS chromatogram of extract of microplastic samples collected during the April 2017 campaign.

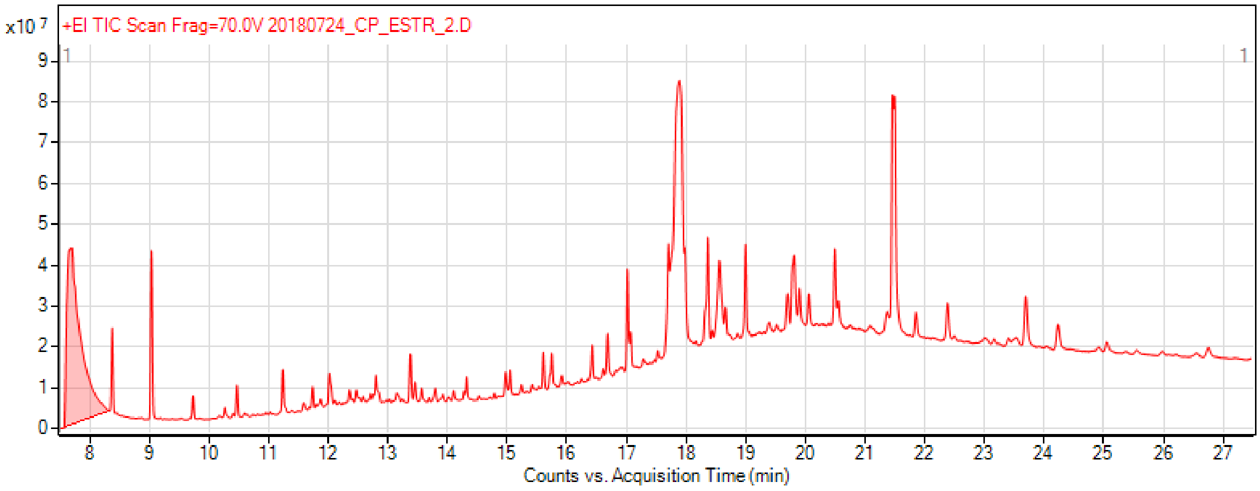

Figure S6. HRGC–MS chromatogram of extract of microplastic samples collected during the February 2017 campaign.

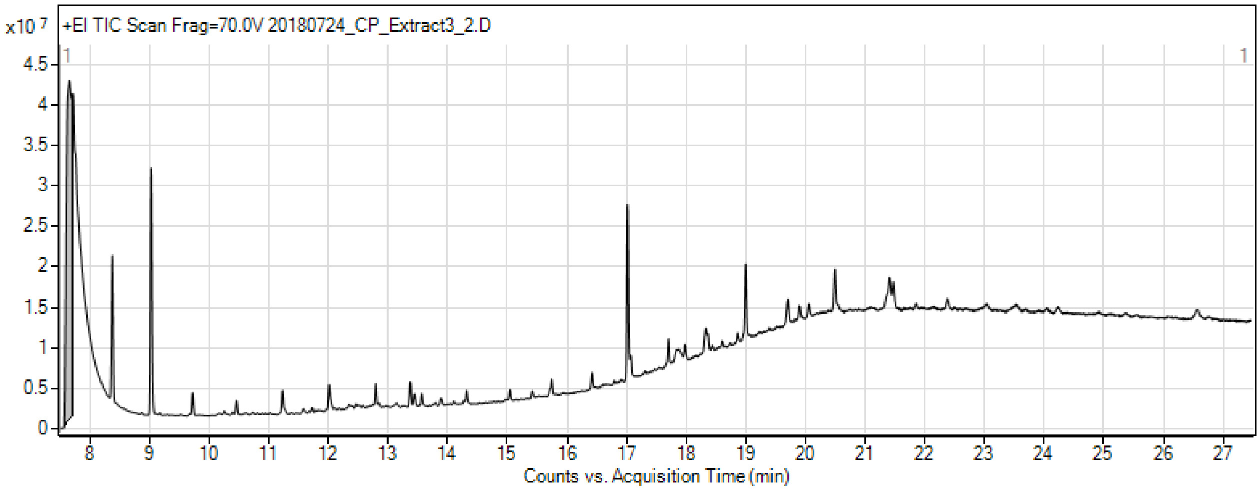

Figure S7. HRGC–MS chromatogram of extract of microplastic samples collected during the December 2017 campaign.

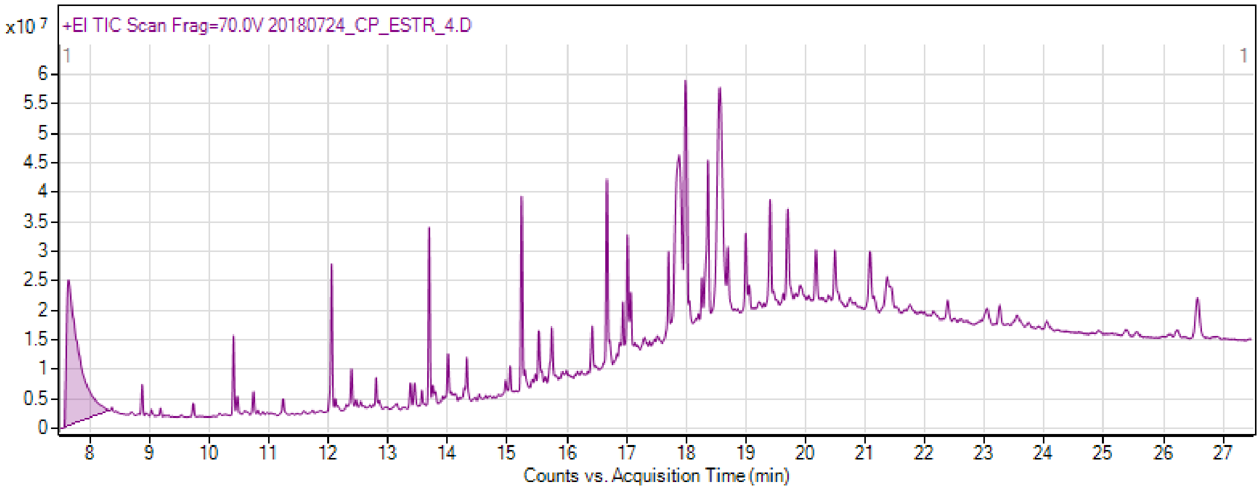

Figure S8. HRGC–MS chromatogram of extract of microplastic samples collected during the May 2018 campaign.

To further confirm the identity of each deconvoluted peak, we used accurate mass information to propose, for the major ions of the spectra of a subset of the hypothetic compounds, a molecular formula in order to calculate a mass error associated. Mass errors were calculated as follows:

$$[(\text{Measured mass} - \text{Calculated mass})/\text{Calculated mass}] \times 1000 \quad (2)$$

Fifteen unknown plastic related compounds, identified for comparison by spectra of NIST 17 library with a match factor ≥85%, were further confirmed on the basis of accurate mass measurements calculating mass errors related to the major ions observed for each compound (Table S12).

Table S12. Accurate mass measurements and elemental compositions of compounds found on microplastic and their product ions using HRGC-MS analysis.

Structure	Name	Formula	Score	Major Ions (m/z)	Elemental Composition	Calculated Mass (m/z)	Error (ppm)
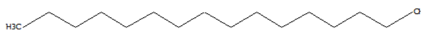	Hexadecane	$C_{16}H_{34}$	96.33	57.0707	C_4H_9	57.0699	−14.43
				43.0546	C_3H_7	43.0542	−8.67
				41.0390	C_3H_5	41.0386	−10.32
				71.0852	C_5H_{11}	71.0855	4.6
				55.0542	C_4H_7	55.0542	0.48
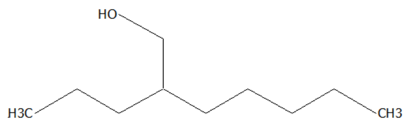	1-Heptanol, 2.propyl-	$C_{10}H_{22}O$	95.9	43.0546	C_3H_7	43.0542	−8.67
				57.0707	C_4H_9	57.0699	−14.43
				41.0390	C_3H_5	41.0386	−10.32
				71.0852	C_5H_{11}	71.0855	4.6
				55.0542	C_4H_7	55.0542	0.48
	Heptacosane	$C_{27}H_{56}$	94.68	57.0707	C_4H_9	57.0699	−14.43
				43.0546	C_3H_7	43.0542	−8.67
				71.0852	C_5H_{11}	71.0855	4.6
				41.0390	C_3H_5	41.0386	−10.32
				85.1007	C_6H_{13}	85.1012	5.6
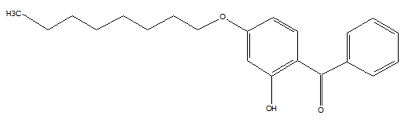	Octabenzone	$C_{21}H_{26}O_3$	94.19	213.0536	$C_{13}H_9O_3$	213.0546	4.79
				214.0594	$C_{13}H_{10}O_3$	214.0624	14.23
				137.1296	$C_{10}H_{17}$	137.1325	20.98
				105.0686	C_8H_9	105.0691	12.15
	Hentatriacontane	$C_{31}H_{64}$	93.29	57.0707	C_4H_9	57.0699	−14.43
				43.0546	C_3H_7	43.0542	−8.67
				71.0852	C_5H_{11}	71.0855	4.6
				85.1007	C_6H_{13}	85.1012	5.6
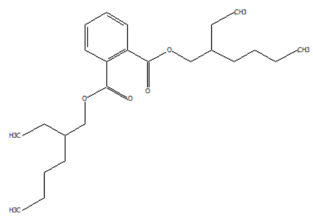	Bis(2-ethylhexyl)phtalate	$C_{24}H_{38}O_4$	92.49	149.0237	$C_8H_5O_3$	149.0233	−2.55
				167.1777	$C_{12}H_{23}$	167.1794	10.33
				57.0707	C_4H_9	57.0699	−14.43
				55.0542	C_4H_7	55.0542	0.48

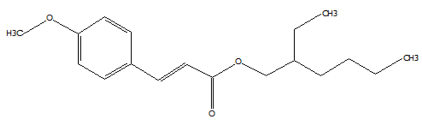	2-Propenoic acid, 3-(4-methoxyphenyl)-, 2-ethylhexyl ester	$C_{18}H_{26}O_3$	92.45	178.0618<br>179.0209<br>161.1306<br>41.0390<br>77.0380	$C_{10}H_{10}O_3$<br>$C_{12}H_3O_2$<br>$C_{12}H_{17}$<br>C_3H_5<br>C_6H_5	178.0624<br>179.0128<br>161.1325<br>41.0386<br>77.0386	3.63<br>−45.49<br>11.65<br>−10.32<br>7.48
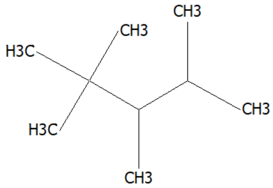	Pentane, 2,2,3,4-tetramethyl-	C_9H_{20}	92.25	57.0707<br>43.0546<br>41.0390<br>56.0620<br>55.0542	C_4H_9<br>C_3H_7<br>C_3H_5<br>C_4H_8<br>C_4H_7	57.0699<br>43.0542<br>41.0386<br>56.0621<br>55.0542	−14.43<br>−8.67<br>−10.32<br>0.92<br>0.48
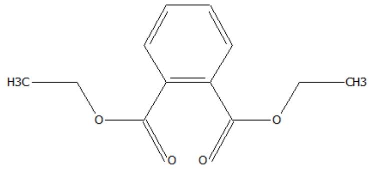	Diethyl phthalate	$C_{12}H_{14}O_4$	88.20	149.0237<br>121.0995<br>65.0382	$C_8H_5O_3$<br>C_9H_{13}<br>C_5H_5	149.0233<br>121.1012<br>65.0386	−2.55<br>13.85<br>5.79
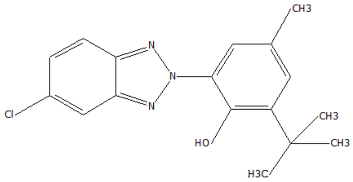	Bumetrizole	$C_{17}H_{18}ClN_3O$	88.01	300.0888<br>91.0541	$C_{16}H_{15}ClN_3O$<br>C_7H_7	300.0898<br>91.0542	3.39<br>1.39
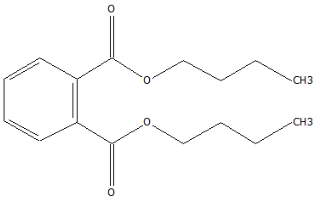	Dibutyl phthalate	$C_{16}H_{22}O_4$	87.09	149.0237<br>76.0304<br>65.0382	$C_8H_5O_3$<br>C_6H_4<br>C_5H_5	149.0233<br>76.0308<br>65.0386	−2.55<br>4.62<br>5.79
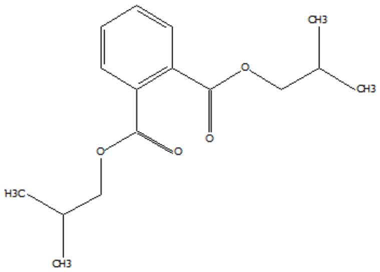	1,2-Benzenedicarboxylic acid, bis(2-methylpropyl) ester	$C_{16}H_{22}O_4$	92	149.0237<br>57.0707<br>104.0599<br>41.0390	$C_8H_5O_3$<br>C_4H_9<br>C_8H_8<br>C_3H_5	149.0233<br>57.0699<br>104.0621<br>41.0386	−2.55<br>−14.43<br>20.68<br>−10.32

	Octacosane	$C_{28}H_{58}$	91.34	85.1007	C_6H_{13}	85.1012	5.6
				43.0546	C_3H_7	43.0542	−8.67
				41.0390	C_3H_5	41.0386	−10.32
				57.0707	C_4H_9	57.0699	−14.43
				55.0542	C_4H_7	55.0542	0.48
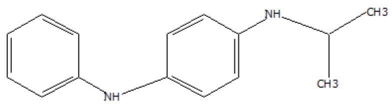	1,4-Benzenediamine, N-(1-methylethyl)-N′-phenyl-	$C_{15}H_{18}N_2$	86.59	226.1453	$C_{15}H_{18}N_2$	224.1465	5.09
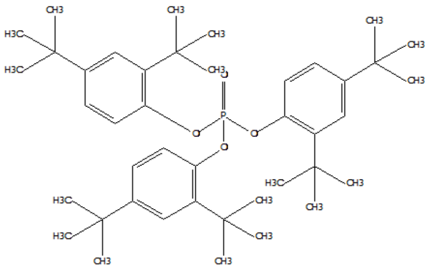	Tris(2,4-di-tert-butylphenyl) phosphate	$C_{42}H_{63}O_4P$	86.12	647.4214	$C_{41}H_{60}O_4P$	647.4224	1.5
				316.1983	$C_{20}H_{29}OP$	316.1951	−10.27
				648.4240	$C_{41}H_{61}O_4P$	648.4302	9.56
				57.0707	C_4H_9	57.0699	−14.43

Table S13. Comparison of high-resolution spectra of 15 hypothetically identified compounds by spectra of NIST 17 library.

Hexadecane

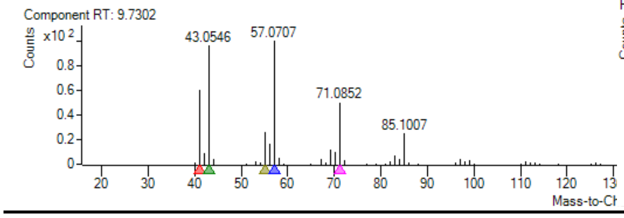

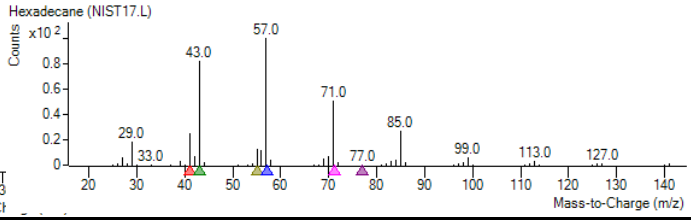

1-Heptanol, 2-propyl-

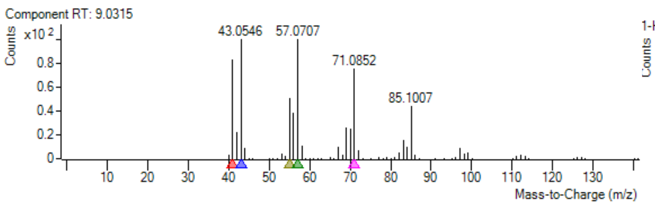

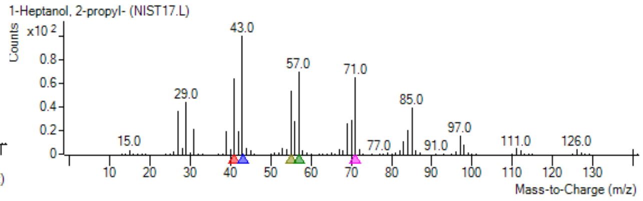

Heptacosane

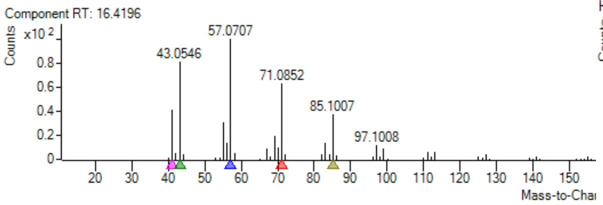

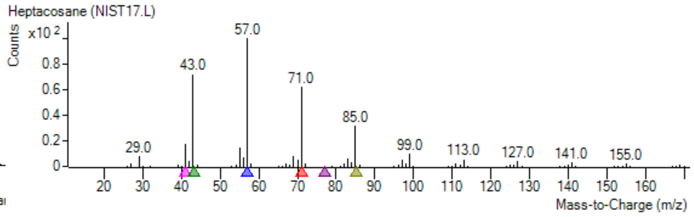

Octabenzone

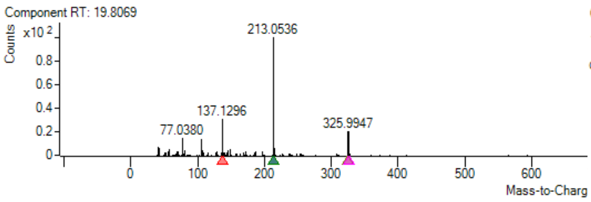

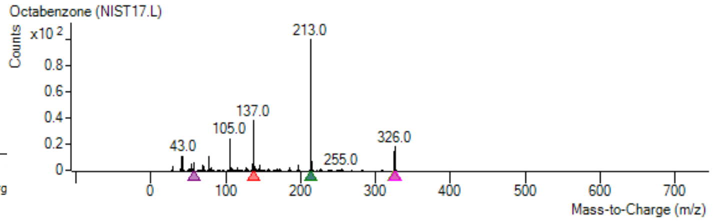

Hentriacontane

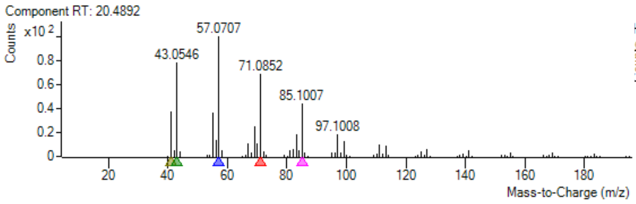

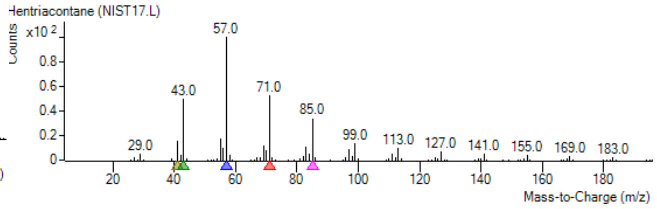

Bis(2-ethylhexyl) phtalate

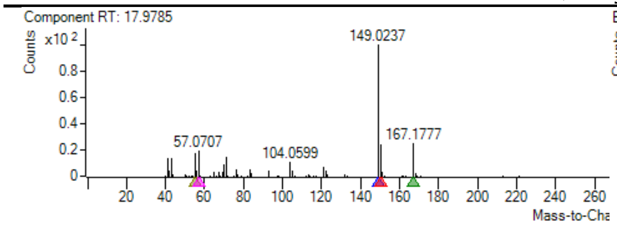

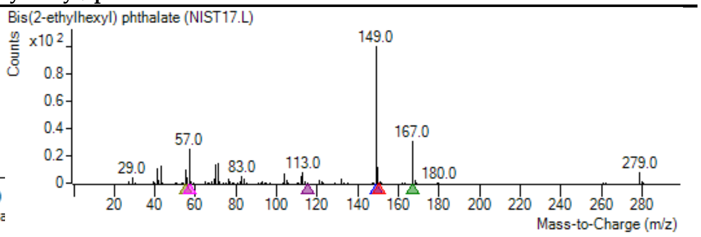

2-Propenoic acid, 3-(4-methoxyphenyl)-, 2-ethylhexyl ester

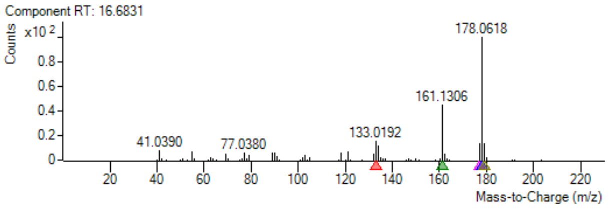

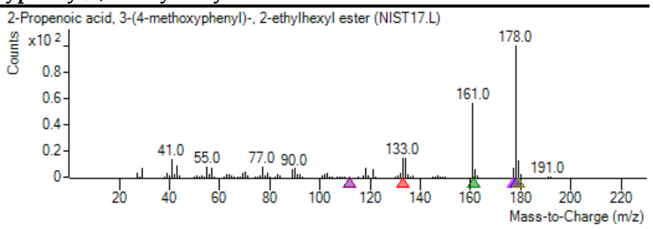

Pentane, 2,2,3,4-tetramethyl-

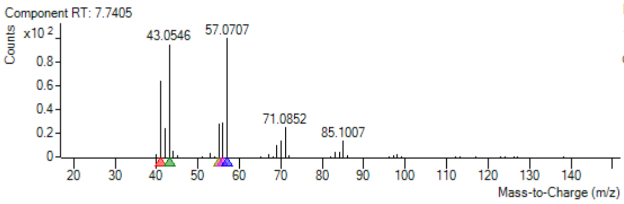

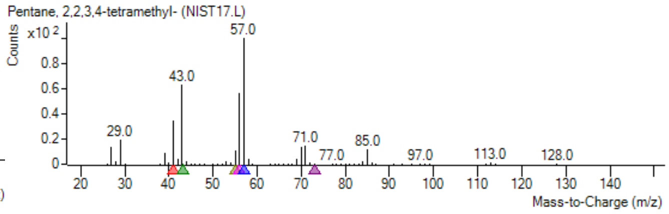

Diethyl phtalate

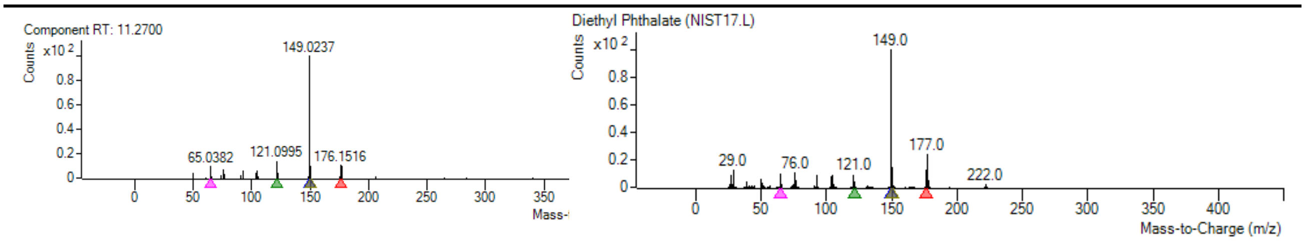

Bumetrizole

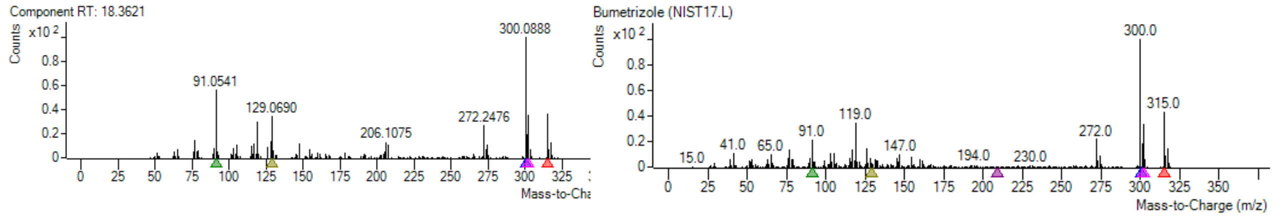

Dibutyl phtalate

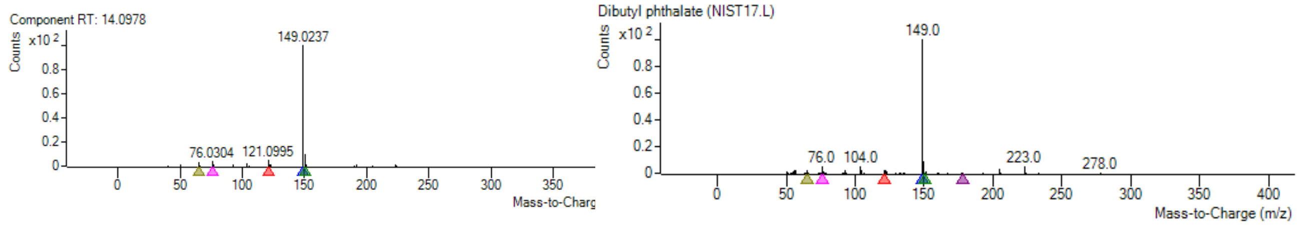

1,2-Benzenedicarboxylic acid, bis(2-methylpropyl) ester

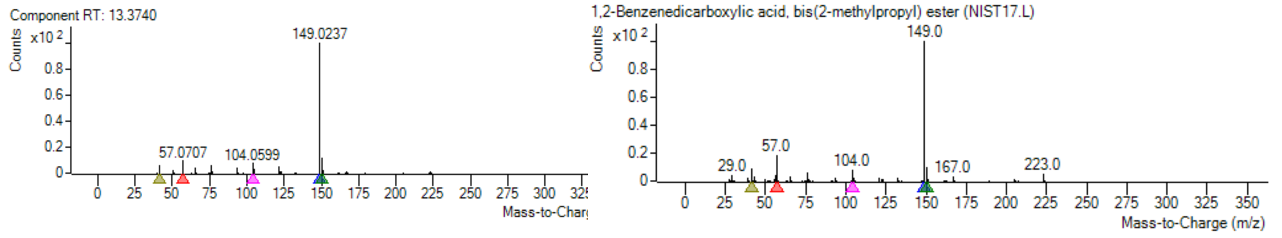

Octacosane

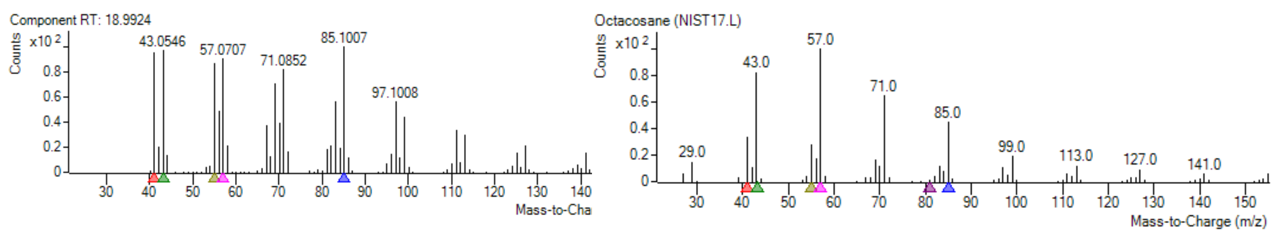

1,4-Benzendiamine, N-(1-methylethyl)-N′-phenyl-

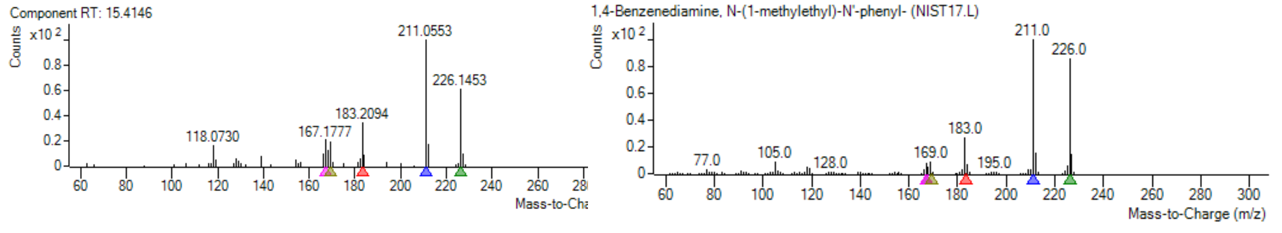

Tris(2,4-di-tert-butylphenyl) phosphate

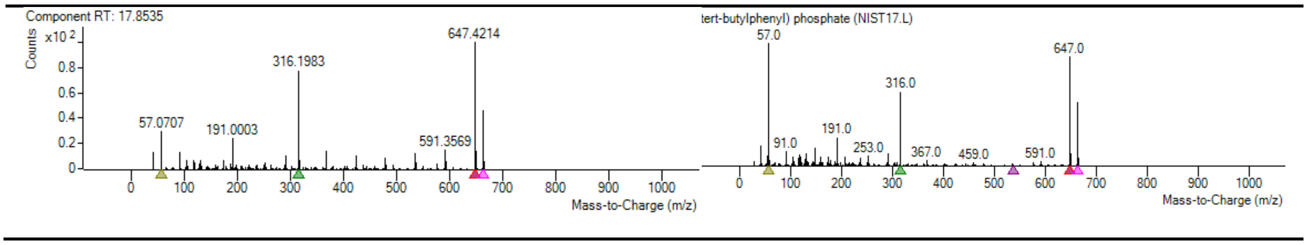
Component RT: 17.8535
Counts x10 2
647.4214
316.1983
57.0707
191.0003
591.3569
Mass-to-Charge (m/z)
tert-butylphenyl) phosphate (NIST17.L)
57.0
647.0
316.0
191.0
91.0
253.0
367.0
459.0
591.0
Mass-to-Charge (m/z)
